# Performance analysis of noninvasive electrophysiological methods for the assessment of diabetic sensorimotor polyneuropathy in clinical research: a systematic review and meta-analysis with trial sequential analysis

**DOI:** 10.1038/s41598-020-78787-0

**Published:** 2020-12-10

**Authors:** Fahmida Haque, Mamun Bin Ibne Reaz, Sawal Hamid Md Ali, Norhana Arsad, Muhammad Enamul Hoque Chowdhury

**Affiliations:** 1 Department of Electrical, Electronic and System Engineering, Universiti Kebangsaan Malaysia, 43600 Bangi, Selangor Malaysia; 2 Department of Electrical Engineering, Qatar University, 2713 Doha, Qatar

**Keywords:** Medical research, Neurology

## Abstract

Despite the availability of various clinical trials that used different diagnostic methods to identify diabetic sensorimotor polyneuropathy (DSPN), no reliable studies that prove the associations among diagnostic parameters from two different methods are available. Statistically significant diagnostic parameters from various methods can help determine if two different methods can be incorporated together for diagnosing DSPN. In this study, a systematic review, meta-analysis, and trial sequential analysis (TSA) were performed to determine the associations among the different parameters from the most commonly used electrophysiological screening methods in clinical research for DSPN, namely, nerve conduction study (NCS), corneal confocal microscopy (CCM), and electromyography (EMG), for different experimental groups. Electronic databases (e.g., Web of Science, PubMed, and Google Scholar) were searched systematically for articles reporting different screening tools for diabetic peripheral neuropathy. A total of 22 studies involving 2394 participants (801 patients with DSPN, 702 controls, and 891 non-DSPN patients) were reviewed systematically. Meta-analysis was performed to determine statistical significance of difference among four NCS parameters, i.e., peroneal motor nerve conduction velocity, peroneal motor nerve amplitude, sural sensory nerve conduction velocity, and sural sensory nerve amplitude (all p < 0.001); among three CCM parameters, including nerve fiber density, nerve branch density, and nerve fiber length (all p < 0.001); and among four EMG parameters, namely, time to peak occurrence (from 0 to 100% of the stance phase) of four lower limb muscles, including the vastus lateralis (p < 0.001), tibialis anterior (p = 0.63), lateral gastrocnemius (p = 0.01), and gastrocnemius medialis (p = 0.004), and the vibration perception threshold (p < 0.001). Moreover, TSA was conducted to estimate the robustness of the meta-analysis. Most of the parameters showed statistical significance between each other, whereas some were statistically nonsignificant. This meta-analysis and TSA concluded that studies including NCS and CCM parameters were conclusive and robust. However, the included studies on EMG were inconclusive, and additional clinical trials are required.

## Introduction

Diabetic sensorimotor polyneuropathy (DSPN) is a common and costly complication that is experienced by patients with diabetes; this complication, which involves disruption in the anatomy of the nerve and blood vessels that subsequently leads to the dysfunction of the motor, sensory, and autonomic nerves, has an estimated prevalence of 50%^1–3^. The variance in attributes and symptoms of nerve injury in patients with DSPN makes diagnostic strategies challenging. DSPN causes dispersed regular and length-dependent damage to peripheral nerves, sensation loss, and foot muscle dysfunction, thus leading to increased healthcare cost and decreased quality of life; it is also an early indicator of nonhealing diabetic wounds, infections, diabetic foot ulcers, amputations, and death^3–5^. Early detection and improved classification tools can allow the correct diagnosis and treatment of DSPN, as well as timely intervention to prevent foot ulceration, amputation, and other diabetic complications, hence reducing the possibilities of mortalities due to DSPN^6–8^.

A large number of specialized screening and diagnostic tests for the assessment of DSPN are available, and in most cases, neurological history, physical examination, and electrophysiological tests are combined for the accurate conventional assessment of DSPN^9,10^. Some of the most common clinical and electrophysiological diagnostic methods for DSPN are vibration sensation with a 128 Hz tuning fork, monofilament test, quantitative sensory testing (QST)^11,12^, skin biopsy^13^, nerve conduction study (NCS)^14^, corneal confocal microscopy (CCM)^15^, and electromyography (EMG)^16^.

Given the lack of reliable estimates for the frequency of DSPN in different populations and the absence of clear diagnostic guidelines^17,18^, different clinical studies have been conducted by using various screening methods to identify DSPN^19–40^. QST, neuropathy disability score, Michigan neuropathy screening method, vibration sensing with a 128 Hz tuning fork, and monofilament test are used for assessing pain, touch, vibration, and temperature sensation loss due to DSPN^9–12^. However, the change in nerve and muscle function due to DSPN and the progression of muscle and nerve dysfunction with the advancement of DSPN cannot be clearly observed by using these methods. Electrophysiological tests, such as NCS, CCM, and EMG, provide information regarding nerve and muscle dysfunction due to DSPN. NCS has been considered as the gold standard for clinical research or trials on patients with DSPN because of its advantages of objectivity, sensitivity, reliability, noninvasiveness, and association with small coefficients of variation^9,14,41^. CCM is a new rapid, regenerable, and noninvasive method for accurately detecting small-fiber neuropathy. This method allows studying the structure of the human cornea in vivo and has immense potential for studying different corneal diseases^9^. Given that small nerve fibers are the first to be damaged due to DSPN, CCM has shown reasonable diagnostic utility for the assessment of small-fiber DSPN^19–31,33,42^. According to the European Federation of Neurological Society guideline in 2005, skin biopsy is one of the most accurate diagnostic methods for small-fiber DSPN; however, this method cannot be advocated for routine use because it is an invasive and highly specialized procedure that requires electron microscopy and professional expertise^43,44^. Diabetic neuropathy leads to the progressive loss of somatosensory sensitivity, especially in the lower limbs; this effect may cause functional gait variations and is predominantly related to reductions in joint movement range and active muscle power and changes in gait mechanics^45^. EMG has been widely used by researchers to observe muscle activities and diagnose DSPN in patients^36–40^. Thus, in this study, the three noninvasive electrophysiological diagnostic methods NCS, CCM, and EMG were considered for meta-analysis and systematic review.

In numerous studies, CCM and NCS have been used together to identify DSPN^19,24,26–28,30^. NCS and EMG^46–50^ are also widely used to diagnose neuromuscular diseases, and their severity such as DSPN. However, the significance of relationships among these different methods and their parameters and threshold values have not been determined uniformly in existing studies. Another major drawback of existing studies is their differences in sample sizes, patient’s characteristics, environments, and diagnostic tools. These factors affect their results and introduce bias. A review of the existing literature revealed the absence of studies with a large sample size from which standardized values of different parameters can be established for these diagnostic tests. An existing clinical study on DSPN with a large sample size can be considered as a reference for understanding the characteristics of different patient groups and the baseline values of different diagnostic parameters for those groups. A meta-analysis can be a very powerful tool for summarizing results from different studies and obtaining conclusive results from different reported studies. To our knowledge, no meta-analysis has been conducted on DSPN screening methods to observe the baseline values for diagnostic parameters and to identify the statistical significance between screening parameters. Therefore, this study aimed to conduct a meta-analysis to assess the significance among the different parameters of the most commonly used electrophysiological screening methods for DSPN (NCS, CCM, and EMG) in clinical research. It also aimed to summarize the results of different studies to produce a single estimate of the major effect with enhanced accuracy for the different diagnostic parameters of patients with DSPN when compared with those of healthy controls and diabetic patients without DSPN (non-DSPN). We conducted trial sequential analysis (TSA) to validate the meta-analysis and to identify the effect of the included studies for different DSPN diagnostic methods.

## Methods

### Literature search strategy

Electronic databases (Web of Science, PubMed, Scopus, and Google Scholar) were searched systematically for articles published between January 2007 and June 2019. The search strategy was based on a combination of terms: (1) diabetic neuropathy. (2) Electromyography* AND diabetic neuropathy. (3) Nerve conduction studies AND diabetic neuropathy. (4) Corneal confocal microscopy AND diabetic neuropathy. The following inclusion criteria were adopted: (i) published between January 2007 and June 2019; (ii) included at least 10 adult patients with DSPN; (iii) reported any of the three diagnostic methods for DSPN, namely, NCS, CCM or EMG; (iv) reported the values of at least two diagnostic parameters out of the four NCS parameters, three CCM parameters, and four EMG parameters selected for this study. Exclusion criteria included abstracts from conferences, articles that did not report diagnostic parameters, diagnostic parameter data presented graphically that could not be retrieved, case reports, comments, and reviews.

### Selection of studies and data extraction

**Figure 1:**
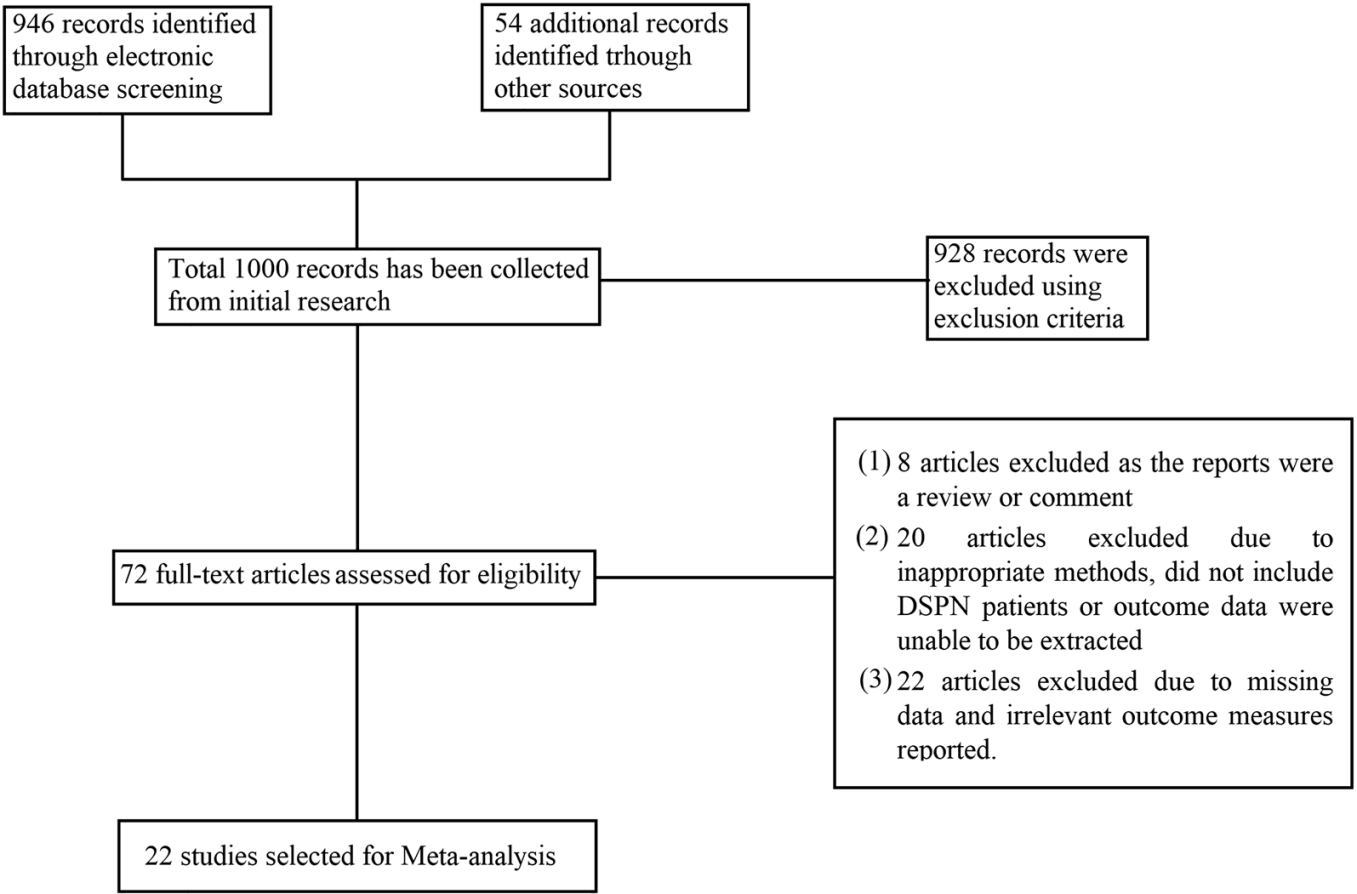
Study selection process for meta-analysis.

The titles and abstracts retrieved from the initial database search were screened on the basis of the literature search strategy. The full text was reviewed for articles that remained relevant after the initial screening on the basis of inclusion criteria. All studies meeting the exclusion criteria were removed from the review. The following data were extracted from the eligible articles: study details (title, author list, year of publication, journal of publication, citation, method used, major finding, and a short summary); patient characteristics (number of patients, experimental group, age, sex, DM duration, HbA1c [%], and body mass index); and diagnostic parameters for NCS, namely, peroneal motor nerve conduction velocity (PMNCV), peroneal motor nerve amplitude (PMNamp), sural sensory nerve conduction velocity (SSNCV), and sural sensory nerve amplitude (SSNamp); parameters for CCM, including nerve fiber density (NFD), nerve branch density (NBD), nerve fiber length (NFL); and parameters for EMG, including time to peak occurrence (from 0 to 100% of the stance phase) for the vastus lateralis (VL), tibialis anterior (TA), lateral gastrocnemius (LG), gastrocnemius medialis (GM) muscles, and vibration perception threshold (VPT). Extracted data were recorded in a tabular manner to prepare a summary form for each study. Figure 1 shows the flow chart of the selection of the studies for meta-analysis. The first author was responsible for the study selection, study design, data extraction, and meta-analysis, and all authors were involved in result analysis, data representation, and manuscript preparation.

### Meta-analysis

Data were transformed into standardized units of measure in the form of mean ± standard deviation (SD) for comparison and statistical analysis when possible. Meta-analysis was carried out on individual outcome measures when more than two studies reported the particular individual outcome measure. Statistical significance (p) between different diagnostic parameters was calculated with Student’s *t*-test. Here, p < 0.05 was considered to be statistically significant. The standard mean difference and the corresponding 95% confidence intervals (95% CIs) for all diagnostic parameters were calculated. Heterogeneity was measured by using the I^2^ statistic, and I^2^ > 50% was considered significantly heterogeneous. All the statistical analyses were performed with Minitab version 18.0 software (Minitab LLC, State College, Pennsylvania, USA). Meta-analysis was performed by using the Review Manager (RevMan) 5.3 computer program (Copenhagen: The Nordic Cochrane Centre, The Cochrane Collaboration, 2014).

### Trial sequential analysis (TSA)

A meta-analysis with a small sample size may lead to a false negative or positive conclusion even with a statistical significance. TSA (TSA, version 0.9 beta, http://www.ctu.dk/tsa/) (Copenhagen Trial Unit, Centre for Clinical Intervention Research, Rigshospitalet, Copenhagen, Denmark)^51^ was performed to avoid that type of error in this meta-analysis and to validate the conclusion from the meta-analysis. TSA uses a combination of statistical analyses to identify the required information size (RIS), which helps evaluate if sufficient information has been included and whether the outcomes of a meta-analysis are reliable or not^51^. A decision is considered conclusive from the meta-analysis if the Z-curve crosses the TSA boundary or enters the futility area, indicating that further studies are not required in that meta-analysis or else the meta-analysis is inconclusive and additional studies should be required. The RIS was calculated on the basis of an alpha risk of 5% error, 90% statistical power, and a two-sided boundary type for continuous data.

## Results

### Study selection

Overall, 1000 unique records were originally identified. However, 928 articles were excluded for a variety of reasons, such as unsuitable study design, inappropriate comparison groups, undesired diagnostic methods, missing data, irrelevant data, or inability to extract data. Thus, 72 articles were considered as potentially relevant, and their full texts were retrieved. Upon reviewing the full texts, 50 studies were excluded because (1) the report was a review or commentary (n = 8); (2) the study did not include patients with DSPN or outcome data could not be extracted (n = 20); and (3) the study did not include the parameters desired for the meta-analysis (n = 22). Finally, 22 remaining studies were eligible for inclusion. Several studies used CCM and NCS methods^19,24,26–28,30^. A total of 13 studies on CCM^19–31^, 10 on NCS^19,24,26–28,30,32–35^, and five on EMG^36–40^ were selected for this analysis.

### Participants’ characteristics

Table 1 displays a summary of the characteristics and sociodemographic variables of the participants in the included studies. The 22 studies included for meta-analysis had 2394 participants (801 patients with DSPN, 702 controls, and 891 non-DSPN patients) in total from three different experimental groups^19–40^. The mean group size was 44.21 and ranged from 10 to 164 participants. The age range of participants in the control, non-DSPN, and DSPN groups from the included studies were 47.76 ± 15.28, 48.34 ± 15.56, and 55.40 ± 12.24 years, respectively. The mean diabetes duration in the DSPN group was higher than that in the non-DSPN group. Table 1 shows that patients with DSPN had higher HbA1c (%) than other participants, and no significance difference in BMI (kg/m^2^) between the control and non-DSPN groups were observed.

**Table 1:** Characteristics of included studies^19–40^.

Study	Diagnostic Method	Group	N	Age (years)	Sex (M/F)	Duration of diabetes (years)	HbA1c (%)	BMI (kg/m^*2*^*)*
Ahmed et al.^19^	NCS CCM	Control	64	38.9 ± 17.6	30/34	–	5.5 ± 0.4	24.7 ± 4.6
		No-DSPN	56	34.9 ± 14.8	27/29	17.6 ± 14.0	7.4 ± 1.3	25.3 ± 4.4
		DSPN	33	50.0 ± 14.3	16/17	31.4 ± 13.5	8.7 ± 2.1	28.9 ± 5.0
Akashi et al.^38^	EMG	Control	16	51.1 ± 8.3	8/8	–	–	23.9 ± 2.9
		DSPN	19	57.6 ± 8.5	11/8	12.6 ± 5.3	–	26.6 ± 4.2
		DSPN-U	10	53.8 ± 7.9	5/5	16.4 ± 8.5	–	27.8 ± 4.6
Alam et al.^33^	NCS CCM	Control	27	41 ± 14.9	16/11	-	5.5 ± 0.31	26.9 ± 4.0
		No- DSPN	30	38.8 ± 12.5	13/14	17.2 ± 12.0	7.7 ± 1.92	26.3 ± 4.4
		DSPN	31	53.3 ± 11.9	19/12	37.2 ± 13.1	8.6 ± 1.55	27.2 ± 4.2
Chen et al.^30^	NCS CCM	Control	26	44 ± 15	–		5.5 ± 0.3	26.8 ± 4.0
		No-DSPN	46	44 ± 13	–	23 ± 15	8.2 ± 1.4	26.4 ± 4.5
		DSPN	17	59 ± 11	–	39 ± 14	8.5 ± 1.3	27.5 ± 3.5
Edwards et al.^20^	CCM	Control	61	52 ± 14	27/34	–	5.4 ± 0.3	26.1 ± 5.1
		No-DSPN	143	48 ± 16	66/77	14 ± 12	7.8 ± 1.2	27.6 ± 5.3
		DSPN	88	58 ± 9	57/31	23 ± 14	8.2 ± 1.7	30.7 ± 7.2
Gomes et al.^39^	EMG	Control	23	55 ± 8	9/14	–	–	–
		DSPN	23	56 ± 8	9/14	14.4 ± 6.5	–	–
Hertz et al.^21^	CCM	Control	20	41.4 ± 17.3	5/15	—	5.5 ± 0.4	–
		DM-1	26	42.8 ± 16.9	18/8	22.7 ± 16.4	8.0 ± 1.9	–
Hussain et al.^34^	NCS	No-DSPN	22	51.90 ± 6.46	10/12	2 ± 1.7	6.67 ± 0.98	25.38 ± 2.93
		DSPN	64	54.68 ± 7.59	28/36	7.59 ± 4.77	7.9 ± 2.2	25.63 ± 3.19
Li et al.^31^	NCS CCM	Control	24	68.63 ± 5.19	9/15	–	5.88 ± 0.82	25.41 ± 3.57
		No-DSPN	49	67.12 ± 6.01	18/30	9.79 ± 7.09	7.07 ± 0.96	24.56 ± 2.93
		DSPN	79	70.15 ± 7.34	46/33	12.58 ± 7.28	7.94 ± 1.86	25.38 ± 3.40
Malik et al.^22^	CCM	Control	18	57.8 ± 11.5	–	–	< 6.5	–
		DSPN	18	57.82 ± 11.90	–	22.93 ± 11.79	8.07 ± 1.34	–
Mehra et al.^23^	CCM	Control	15	46 ± 3	–	–	–	–
		DSPN	20	41 ± 1	20/0	27 ± 2	8.9 ± 1.4	–
Petropoulos et al.^24^	NCS CCM	Control	55	51.7 ± 11.4	28/27	–	5.5 ± 0.3	25.6 ± 4.6
		No-DSPN	86	50.4 ± 14.1	108/78	24.2 ± 21.2	7.7 ± 1.6	27.2 ± 5.2
		DSPN	100			34.4 ± 17.3	7.9 ± 1.6	27.6 ± 5.8
Pritchard et al.^25^	CCM	Control	154	46 ± 15	70/84	–	5.5 ± 0.3	28 ± 6
		No-DSPN	168	43 ± 16	85/83	20 ± 15	8.0 ± 1.2	26 ± 4
		DSPN	74	57 ± 11	41/33	34 ± 16	8.6 ± 1.8	26 ± 5
Quattrini et al.^26^	CCM	Control	15	55.0 ± 18.5	6/9	–	–	–
		No-DSPN	10	53.5 ± 10.2	6/4	16.7 ± 4.44	7.16 ± 0.40	–
		DSPN	44	59.05 ± 3.18	36/8	21.22 ± 4.24	8.01 ± 0.42	–
Sivaskandarajah et al.^27^	CCM NCS	Control	64	38.3 ± 16.4	34/30	–	5.6 ± 0.4	24.7 ± 5.0
		No-DSPN	63	32.7 ± 13.6	29/34	17.3 ± 12.2	7.5 ± 1.2	24.9 ± 3.6
		DSPN	33	48.5 ± 13.7	14/19	32.3 ± 13.1	8.4 ± 1.6	27.7 ± 6.1
De Souza et al.^32^	NCS	Control	51	38.5 ± 14.2	24/27	–	≤ 6%	–
		No-DSPN	50	46.4 ± 16.5	25/25	9.3 ± 15.1	8.1	–
		DSPN	52	57.3 ± 11.7	27/25	10.9 ± 8	9.2	–
Sacco et al.^36^	EMG	Control	21	50.9 ± 7.3	–	–	–	24.3 ± 2.6
		DSPN	24	55.2 ± 7.9	–	–	–	27.0 ± 4.4
Sawacha et al.^37^	EMG	Control	10	61.2 ± 5.07	–	–	–	24.4 ± 2.8
		No-DSPN	20	56.53 ± 13.29	–	23.3 ± 13.7	–	26.4 ± 2.5
		DSPN	20	61.2 ± 7.7	–	13 ± 6.5	–	26.8 ± 3.4
Tavakoli et al.^28^	NCS CCM	Control	17	55 ± 4.8	8/9	–	< 6.5	–
		No-DSPN	34	55 ± 1.9	19/15	10.7 ± 1.82	8.1 ± 0.27	–
		DSPN	67	58.87 ± 2.46	54/13	17.03 ± 3.02	8.10 ± 0.38	–
Tavakoli et al.^29^	CCM	Control	18	57.0 ± 3.0	10/8	–	5.7 ± 0.1	–
		DSPN	25	52.0 ± 2.0	20/5	26.5 ± 2.5	8.1 ± 0.3	–
Weisman et al.^35^	NCS	No-DSPN	84	56 ± 9	48/36	10 ± 11	7.8 ± 1.7	29.9 ± 5.15
		DSPN	25	55 ± 10	16/9	10 ± 7	9.0 ± 1.7	28.9 ± 5.82
Watari et al.^40^	EMG	Control	30	54.1 ± 7.5	14/16	–	–	25.7 ± 3.9
		No-DSPN	43	56.7 ± 6.8	25/18	8.1 ± 7.2	–	28.4 ± 3.9
		DSPN	74	56.7 ± 6.0	45/29	13.4 ± 7.1		28.8 ± 4.2
Total		Control	702	47.76 ± 15.28	–	–	5.53 ± 0.38	25.21 ± 6.85
		No-DSPN	891	48.34 ± 15.56	–	15.69 ± 14.41	7.77 ± 1.29	26.80 ± 4.68
		DSPN	801	55.40 ± 12.24	–	23.85 ± 15.43	8.29 ± 1.64	27.43 ± 5.56

### Nerve conduction study (NCS)

NCS has been used as a standardized clinical test for diagnosing DSPN and for validating other diagnostic methods. In 10 studies^19,24,26–28,30,32–35^, a total of 1231 participants (471 patients with DSPN, 292 controls, and 468 non-DSPN patients) underwent NCS. In all the included studies, the main observed parameters were the nerve conduction velocity (NCV) and nerve amplitude (Namp) for peroneal motor (PM) and sural sensory (SS) nerves. All the NCS parameters are listed in Table 2. However, median nerve^32^, ulnar nerve^32,34^, and tibial nerve^35^ parameters were reported in a few studies which were insufficient for a conclusive meta-analysis. Thus, only PM and SS parameters were considered for meta-analysis. Quattrini et al.^26^ reported the NCS results for five different classes (control, non-DSPN, mild DSPN, moderate DSPN, and severe DSPN). Given that only three experimental groups are considered in this study, the three reported^26^ DSPN severity groups (mild, moderate, and severe) were considered as the DSPN group on the basis of the Cochrane Guidelines for Systematic Review^52^. The PMNCV (m/s) values of patients with DSPN (37.80 ± 6.48) were significantly lower than those of the controls (48.57 ± 3.82) and non-DSPN patients (44.40 ± 4.10). Similarly, the SSNCV (m/s) [49.96 ± 5.10 for Control, 44.57 ± 10.20 for Non-DSPN and 38.61 ± 13.72 for DSPN], SSNamp (μV) [18.67 ± 7.91 for Control, 11.00 ± 6.66 for Non-DSPN, 5.88 ± 4.99 for DSPN] , PMNamp (μV) [5.90 ± 2.25 for control, 5.10 ± 3.77 for Non-DSPN, 2.52 ± 1.95 for DSPN] values of the DSPN group were significantly reduced compared with those of other two experimental groups. A few studies have considered VPT^24,28,30,33^ alongside NCS to observe the change in the vibration sensation of patients with DSPN. These studies showed that VPT (V) values drastically increased for the DSPN group (23.53 ± 11.75) but not for the control (6.51 ± 4.66) or non-DSPN (8.83 ± 5.55) groups (Table 3). All of the NCS parameters of the DSPN groups were significantly reduced in comparison with those of the other two experimental groups, and VPT (V) values drastically increased for DSPN but not for the control or non-DSPN groups.

**Table 2:** NCS Parameter from studies^19,24,26–28,30,32–35^.

Study	Group	N	Peroneal motor nerve conduction velocity (PMNCV) (m/s)	Sural sensory nerve conduction velocity (SSNCV) (m/s)	Sural sensory nerve amplitude (SSNamp) (μV)	Peroneal motor nerve amplitude (PMNamp) (μV)
Ahmed et al.^19^	Control	64	48 ± 3	51 ± 5	18 ± 8	6 ± 2
	No-DSPN	56	43 ± 3	46 ± 4	11 ± 5	6 ± 2
	DSPN	33	36 ± 5	40 ± 3	2 ± 2	2 ± 1
Petropoulos et al.^24^	Control	55	48.8 ± 3.3	51.0 ± 4.8	20.0 ± 9.7	5.2 ± 1.8
	No-DSPN	86	43.7 ± 4.7	46.4 ± 5.8	12.5 ± 7.8	4.5 ± 3.2
	DSPN	100	39.2 ± 6.1	42.2 ± 6.4	6.5 ± 6.6	2.4 ± 2.1
Quattrini et al.^26^	Control	15	45.71 ± 0.99	46.52 ± 1.87	20.25 ± 3.76	4.27 ± 0.64
	No-DSPN	10	44.15 ± 0.79	42.99 ± 1.42	14.44 ± 2.36	4.26 ± 0.59
	DSPN	44	37.36 ± 3.72	38.67 ± 2.99	4.94 ± 1.62	1.99 ± 0.92
Sivaskandarajah et al.^27^	Control	64	48.0 ± 3.5	50.8 ± 4.5	17.5 ± 8.5	6.4 ± 2.3
	No-DSPN	63	43.9 ± 2.3	46.4 ± 4.3	11.9 ± 5.1	5.9 ± 2.2
	DSPN	33	35.9 ± 8.0	35.2 ± 13.7	3.1 ± 2.1	2.6 ± 1.7
Tavakoli et al.^28^	Control	17	49.26 ± 1.63	47.85 ± 2.62	18.62 ± 2.55	5.58 ± 1.02
	No-DSPN	34	44.60 ± 0.65	42.88 ± 0.92	13.74 ± 1.46	3.58 ± 0.28
	DSPN	67	38.37 ± 3.58	39.27 ± 2.60	5.64 ± 1.81	1.79 ± 0.50
Chen et al.^30^	Control	26	49.1 ± 3.4	50.9 ± 3.9	19.7 ± 8.3	6 ± 2.4
	No-DSPN	46	43.9 ± 3.1	45.3 ± 5.2	12.5 ± 6.9	6.0 ± 8.3
	DSPN	17	31.0 ± 9.5	37.8 ± 6.8	4.3 ± 3.5	1.6 ± 1.6
Souza et al.^32^	Control	51	50.08 ± 5.78	47.71 ± 6.66	18.59 ± 6.70	6.46 ± 2.97
	No-DSPN	50	45.76 ± 6.59	43.83 ± 6.13	14.29 ± 5.66	4.92 ± 3.40
	DSPN	52	38.9 ± 8.59	43.54 ± 11.86	10.59 ± 5.77	3.48 ± 2.42
Alam et al.^33^	Control	27	49.2 ± 3.7	50.6 ± 2.0	20.2 ± 8.8	6.1 ± 2.4
	No-DSPN	30	45.5 ± 2.2	47.1 ± 4.1	15.1 ± 6.1	7.3 ± 9.7
	DSPN	31	35.4 ± 8.6	18.4 ± 12.2	5.5 ± 4.2	2.4 ± 2.1
Hussain et al.^34^	No-DSPN	22	49.48 ± 4.03	56.39 ± 3.64	–	–
	DSPN	64	38.14 ± 7.47	49.84 ± 7.62	–	–
Weisman et al.^35^	No-DSPN	84	45.0 ± 3.26	47.2 ± 5.04	9.6 ± 5.55	6.37 ± 2.58
	DSPN	25	41.2 ± 3.6	42.2 ± 5.10	5.74 ± 3.99	5.08 ± 2.95
Total	Control	292	48.57 ± 3.82	49.96 ± 5.10	18.67 ± 7.91	5.90 ± 2.25
	No-DSPN	468	44.40 ± 4.10	44.57 ± 10.20	11.00 ± 6.66	5.10 ± 3.77
	DSPN	471	37.80 ± 6.48	38.61 ± 13.72	5.88 ± 4.99	2.52 ± 1.95

**Table 3:** VPT from studies^24,28,30,33^.

Study	Group	N	Vibration perception threshold (VPT) (V)
Petropoulos et al.^24^	Control	55	5.8 ± 4.6
	No-DSPN	86	9.2 ± 6.5
	DSPN	100	22.3 ± 12.6
Tavakoli et al.^28^	Control	17	9.58 ± 0.93
	No-DSPN	34	9.56 ± 0.84
	DSPN	67	24.93 ± 9.82
Chen et al.^30^	Control	26	6 ± 5.5
	No-DSPN	46	7.6 ± 5.5
	DSPN	17	25.2 ± 13.4
Alam et al.^33^	Control	27	5.3 ± 4.1
	No-DSPN	30	5.6 ± 2.5
	DSPN	31	18.4 ± 12.2
Total	Control	125	6.51 ± 4.66
	No-DSPN	196	8.83 ± 5.55
	DSPN	215	23.53 ± 11.75

### Corneal confocal microscopy (CCM)

CCM is another commonly used noninvasive method in clinical studies on DSPN. The main corneal nerve parameters studied with CCM include NFD, NBD, and NFL. Recent studies have assessed the screening and monitoring of DPN in clinical studies by using CCM^19–31^. According to Akashi et al.^19^, the NFL parameter can identify patients with DSPN more accurately then the other parameters. Most studies stated that CCM^19–31^ parameters progressively decrease with the increasing severity of neuropathy. Our meta-analysis found similar results. Few studies^24,30,31^ have compared manual CCM parameters with automated CCM measurements. Automated corneal nerve fiber measurements are slightly lower than corresponding manual measurements. Thus, in this meta-analysis, only manual CCM was considered for these studies^24,30,31^. A total of 13 studies^19–31^ involving 1830 participants (612 patients with DSPN, 551controls, and 667 non-DSPN patients) were included. The measured manual CCM parameters, namely, NFD, NFL, and NBD, from these studies are listed in Table 4. The average of each CCM parameter for the control, non-DSPN, and DSPN groups was determined. The NFL (no./mm^2^) in the DSPN group (11.76 ± 6.65) was significantly lower than that in the control (19.94 ± 6.64) and non-DSPN (17.72 ± 5.51) groups. The mean NFD (fiber/mm^2^) values for the control, non-DSPN, and DSPN groups were 41.14 ± 9.91, 33.70 ± 9.97, and 24.60 ± 10.18, respectively. The NBD (branches/mm^2^) in the DSPN group (30.91 ± 27.24) was significantly lower than that in the control (63.66 ± 39.12) and non-DSPN (50.92 ± 30.72) experimental groups. The CCM parameter values of patients with DSPN had reduced compared with those of the non-DSPN and control groups.

**Table 4:** CCM Parameter from studies^19–31^.

Study	Group	N	Corneal nerve fiber length (NFL) (mm/mm^2^)		Corneal nerve fiber density (NFD) (fibers/mm^2^)		Corneal branch density (NBD) (branches/mm^2^)	
Ahmed et al.^19^	Control	64	18.4 ± 4.4		43 ± 11		35 ± 14	
	No-DSPN	56	16.7 ± 4.3		39 ± 10		29 ± 16	
	DSPN	33	11.1 ± 3.6		28 ± 9		17 ± 12	
Edwards et al.^20^	Control	61	20 ± 1		–		80 ± 8	
	No-DSPN	143	18.5 ± 0.5		–		69 ± 2.5	
	DSPN	88	16 ± 1		–		57.5 ± 5	
Hertz et al.^21^	Control	20	16.15 ± 4.13		31.9 ± 9.4		37.2 ± 17.7	
	No-DSPN	12	17.12 ± 3.89		36.27 ± 5.7		29.0 ± 12. 7	
	DSPN	14	12.22 ± 4.23		29.64 ± 12.07		25.86 ± 23.76	
Malik et al.^22^	Control	18	13.5 ± 0.3		44.5 ± 14.1		78.9 ± 30.4	
	DSPN	18	6.99 ± 2.21		26 ± 14.99		26.31 ± 19.51	
Mehra et al.^23^	Control	15	9.69 ± 0.7		42.04 ± 3.2		26.73 ± 2.5	
	DSPN	20	2.23 ± 0.28		13.88 ± 2.1		4.04 ± 1.5	
Petropoulos et al.^24^	Control	55	26.4 ± 5.6^M^	21.2 ± 3.5^A^	37.2 ± 6.7^M^	30.0 ± 6.9 ^A^	92.7 ± 38.6^M^	50.4 ± 24.7 ^A^
	No-DSPN	86	20.3 ± 6.7^M^	17.1 ± 4.5 ^A^	26.7 ± 8.5^M^	20.1 ± 8.7 ^A^	54.9 ± 35.7^M^	31.4 ± 25.6 ^A^
	DSPN	100	16.7 ± 7.6^M^	13.7 ± 5.2 ^A^	20.5 ± 9.5 ^M^	14.4 ± 8.9 ^A^	48.7 ± 33.2^M^	20.1 ± 18.7 ^A^
Pritchard et al.^25^	Control	154	23.2 ± 6.3		–		83.5 ± 45.8	
	No-DSPN	168	19.1 ± 5.8		–		61.7 ± 37.2	
	DSPN	74	14.0 ± 6.4		–		40.1 ± 32.1	
Quattrini et al.^26^	Control	15	6.14 ± 1.22	42.10 ± 4.31^I^ [μm]	43.20 ± 5.05	11.21 ± 0.84^I^ (no/mm)	27.39 ± 3.31	139.66 ± 23.42^I^
	No-DSPN	10	3.97 ± 0.80	32.64 ± 2.78 ^I^ [μm]	29.05 ± 3.07	7.22 ± 1.04^I^ (no/mm)	6.87 ± 1.60	44.99 ± 8.93^I^
	DSPN	44	3.75 ± 3.71	28.61 ± 10.20^I^ [μm]	22.12 ± 6.47	4.90 ± 3.27^I^ (no/mm)	7.25 ± 2.78	31.79 ± 15.25^I^
Sivaskandarajah et al.^27^	Control	64	18.8 ± 4.5		45.3 ± 12.0		39.7 ± 16.9	
	No-DSPN	63	17.1 ± 4.2		42.3 ± 9.4		34.6 ± 19.9	
	DSPN	33	11.6 ± 4.0		29.1 ± 10.4		18.2 ± 13.3	
Tavakoli et al.^28^	Control	17	11.21 ± 0.88		45.60 ± 4.47		25.38 ± 2.99	
	No-DSPN	34	8.05 ± 0.71		31.63 ± 2.33		17.42 ± 2.02	
	DSPN	67	4.37 ± 1.31		23.82 ± 5.67		9.71 ± 4.33	
Tavakoli et al.^29^	Control	18	13.5 ± 0.8		46.0 ± 3.8		35.6 ± 6.7	
	DSPN	25	8.3 ± 0.9		18.8 ± 2.1		6.9 ± 1.5	
Chen et al.^30^	Control	26	26.7 ± 3.7^M^	17.7 ± 2.8^A^	36.8 ± 5.3^M^	31.3 ± 6.5^A^	92.8 ± 36.4^M^	44.6 ± 17.2^A^
	No-DSPN	46	20.2 ± 5.1^M^	13.4 ± 3.3^A^	28.3 ± 7.2^M^	22.6 ± 7.3^A^	56.1 ± 30.3^M^	26.2 ± 15.1^A^
	DSPN	17	14.8 ± 8.3^M^	8.8 ± 4.7^A^	16.9 ± 10.1^M^	13.5 ± 9.1^A^	48.2 ± 32.9^M^	15.4 ± 12.1^A^
Li et al.^31^	Control	24	17.81 ± 3.19^M^	14.66 ± 2.31^A^	35.32 ± 5.55^M^	23.18 ± 5.77^A^	41.48 ± 16.50^M^	36.20 ± 12.87^A^
	No-DSPN	49	15.48 ± 3.66^M^	13.37 ± 3.65^A^	35.68 ± 7.64^M^	18.98 ± 7.21^A^	33.02 ± 17.60^M^	32.96 ± 19.30^A^
	DSPN	79	13.60 ± 4.15^M^	11.92 ± 3.51^A^	33.51 ± 8.96^M^	16.88 ± 7.39^A^	25.03 ± 15.95^M^	23.66 ± 15.60^A^
Total	Control	551	19.94 ± 6.64		41.14 ± 9.91		63.66 ± 39.12	
	No-DSPN	655	17.72 ± 5.51		33.70 ± 9.97		50.92 ± 30.72	
	DSPN	624	11.76 ± 6.65		24.60 ± 10.18		30.91 ± 27.24	

^I^Intra-epidermal nerve fiber (IENF).

^A^Automated IVCCM.

^M^Manual IVCCM; IVCCM: in vivo CCM.

### Electromyography (EMG)

EMG^36–40^ is widely used in different clinical research and trials to diagnose DSPN and observe the biomechanics changes in different muscle activities due to DSPN. EMG activities from lower limb muscles were used in five studies^36–40^ with a total of 333 participants (170 patients with DSPN, 100 controls, and 63 non-DSPN patients). In all the included studies, time to peak occurrence (from 0 to 100% of stance phase) for VL, TA, LG, and GM were used as diagnostic parameters for all three experimental groups (Table 5). The time of muscle peak activity occurrence was longer for VL (12.11 ± 3.42), LG (57.57 ± 12.91), and GM (61.48 ± 5.53) but reduced for TA (4.97 ± 3.37) in the DSPN group compared with those in the control group (9.97 ± 3.36 for VL, 59.15 ± 9.86 for LG and 57.54 ± 6.86 for GM). In the DSPN and non-DSPN groups, meta-analysis suggested a non-significantly longer time for peak occurrence in TA (4.97 ± 3.37, 5.08 ± 2.44) and GM (61.48 ± 5.53, 60.5 ± 5.1) and no difference in VL. Given the absence of studies on the LG muscle in non-DSPN patients, meta-analysis was not possible.

**Table 5:** Time of peak occurrence (%) in different lower limb muscle from EMG^36–40^.

Study	Group	N	Time of peak occurrence (%)			
			Vastus lateralis (VL)	Tibialis anterio (TA)	Lateral gastrocnemius (LG)	Gastrocnemius medialis (GM)
Sacco et al.^36^	Control	21	10.76 ± 2.81	5.46 ± 2.36	64.17 ± 3.92	–
	DSPN	24	14.14 ± 2.35	5.61 ± 2.39	65.29 ± 5.35	–
Sawacha et al.^37^	Control	10	–	9.27 ± 1.63	41.60 ± 2.29	–
	No-DSPN	20	–	6.96 ± 1.10	35.9 ± 1.38	–
	DSPN	20	–	11.71 ± 1.13	38.1 ± 1.66	–
Akashi et al.^38^	Control	16	10.82 ± 3.33	6.05 ± 2.15	63.53 ± 3.65	–
	DSPN	19	11.97 ± 2.31	6.10 ± 1.68	62.84 ± 5.06	–
	DSPN-U	10	14.83 ± 3.53	4.64 ± 1.59	68.00 ± 4.78	–
Gomes et al.^39^	Control	23	9.02 ± 3.90	4.33 ± 1.80	–	54.33 ± 6.18
	DSPN	23	10.37 ± 3.18	3.42 ± 1.73	–	60.16 ± 6.99
Watari et al.^40^	Control	30	9.7 ± 3.2	3.7 ± 2.0	–	60.0 ± 6.4
	No-DSPN	43	12.1 ± 2.3	4.2 ± 2.4	–	60.5 ± 5.1
	DSPN	74	11.66 ± 3.57	3.18 ± 2.32	–	61.89 ± 4.98
Total	Control	100	9.97 ± 3.36	5.15 ± 2.57	59.15 ± 9.86	57.54 ± 6.86
	No-DSPN	63	12.1 ± 2.3	5.08 ± 2.44	–	60.5 ± 5.1
	DSPN	170	12.11 ± 3.42	4.97 ± 3.37	57.57 ± 12.91	61.48 ± 5.53

### Meta-analysis of the diagnostic parameters of DSPN

PMNCV, PMNamp, SSNCV, and SSNamp for NCS; NFD, NBD and NFL for CCM; and time to peak occurrence for the VL, TA, LG, and GM and VTP for EMG were subjected to statistical analysis to find significant differences between different screening variables, which can be considered as a substitute for statistically significant paired parameters. Table S1 shows that that for control group, the SSNamp and NFL (p = 0.45); time to peak occurrence for LG and NFD (p = 0.37); PMNCV and SSNCV (p = 0.82); and PMNamp and VPT (p = 1.00) were not statistically significant. For the non-DSPN experimental groups, PMNCV and SSNCS (p = 0.12); time to peak occurrence for VL and SSNamp (p = 0.71); PMNamp and VPT (p = 0.28); PMNamp and time to peak occurrence for TA (p = 0.16); time to peak occurrence for LG and SSNamp (p = 0.47); and time to peak occurrence for VL and LG (p = 0.47) were not statistically significant (Table S2). For the DSPN experimental group, PMNCV and NFD (p = 0.29); time to peak occurrence for VL and NFL (p = 0.68); VPT and NFD (p = 0.44); VPT and PMNCV (p = 0.16); and SSNamp and time to peak occurrence for TA (p = 0.15) (Table S3) were statistically insignificant.

**Figure 2:**
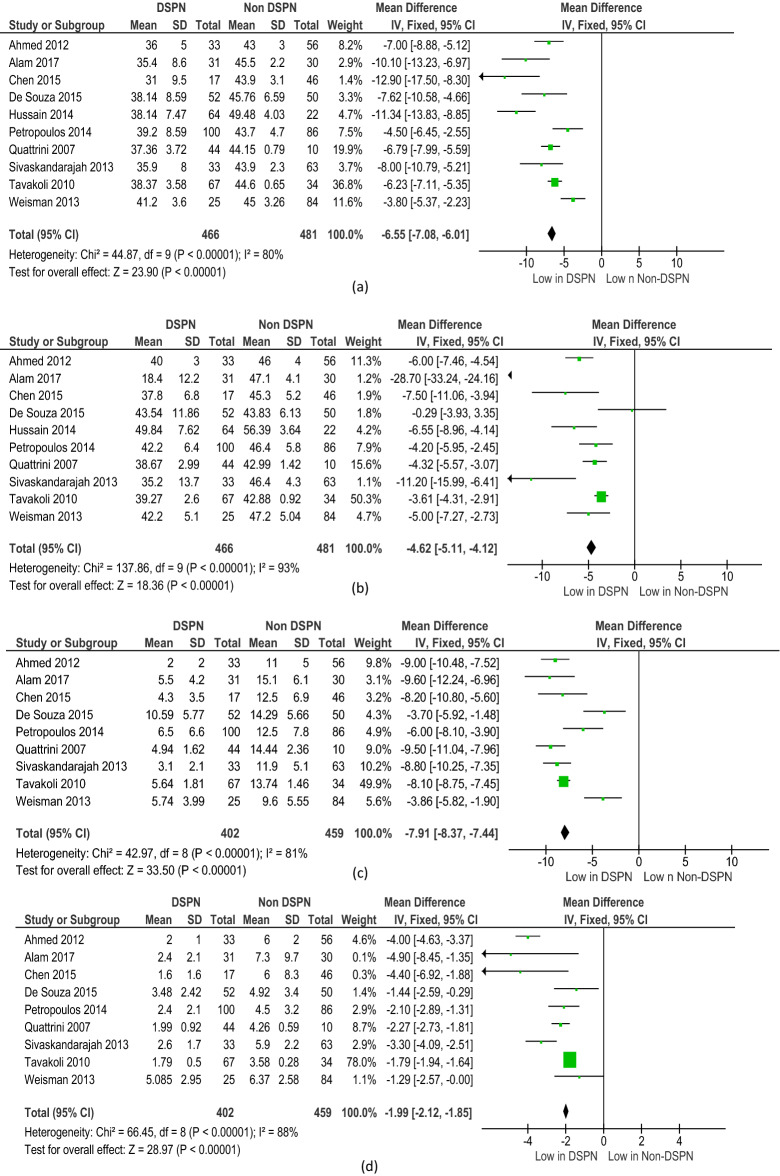
Forest plots for NCS diagnosis parameters comparing DSPN and Non-DSPN groups (**a**) PMNCV, (**b**) SSNCV, (**c**) SSNamp, (**d**) PMNamp.

**Figure 3:**
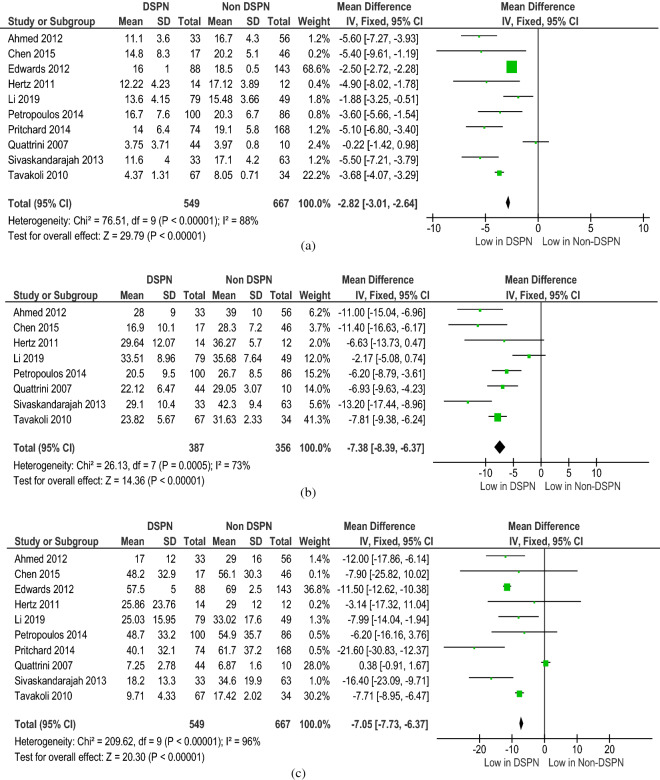
Forest plots for CCM diagnosis parameters comparing DSPN and Non-DSPN groups (**a**) CNFL, (**b**) CNFD, (**c**) CNBD.

**Figure 4:**
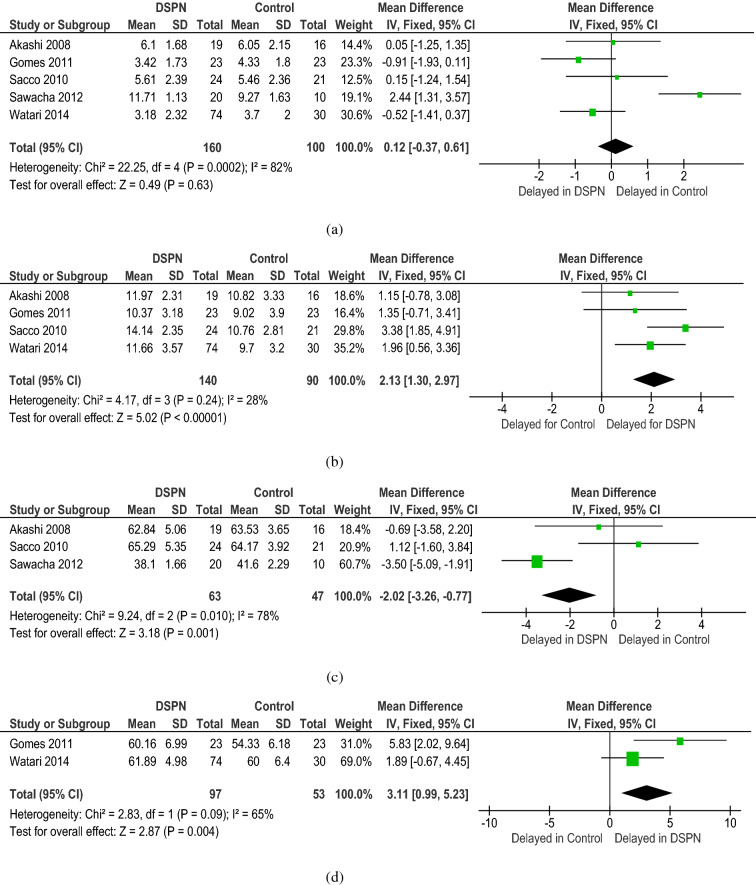
Forest plots for EMG parameters of time for muscle activation peak for four different lower limb muscles (**a**) TA, (**b**) VL, (**c**) LG, (**d**) GM for DSPN and control groups.

**Figure 5:**
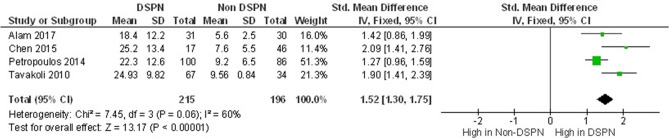
Forest plots for vibration perception threshold (VPT) comparing DSPN and Non-DSPN groups.

All the diagnostic parameters from the included studies were subjected to meta-analysis. The meta-analysis showed good heterogeneity for the following NCS parameters: PMNCV (p < 0.001), SSNCV (p < 0.001), PMNamp (p < 0.001), and SSNamp (p < 0.001) (Fig. 2). The meta-analysis of the CCM parameters of DSPN and non-DSPN groups exhibited good heterogeneity for CNFL (p < 0.001), CNFD (p < 0.001), and CNBD (p < 0.001) (Fig. 3). Given that not all the included studies reported the EMG parameters for non-DSPN patients, meta-analysis was conducted with EMG parameters for the control and DSPN groups. Four different lower limb muscles, namely, the TA (p = 0.63), VL (p < 0.001), LG (p = 0.001), and GM (p = 0.004), were considered for meta-analysis as illustrated in Fig. 4. The time to achieve muscle activation peak for the TA muscle in the DSPN and control groups was not statistically significant, and the meta-analysis indicated low heterogeneity for the VL muscle. GM and LG muscles have moderate and Ta muscles showed good heterogeneity. VPT (p < 0.001) also exhibited moderate heterogeneity (Fig. 5).

### TSA for DSPN diagnostic parameters

TSA was conducted on the NCS and CCM parameters of DSPN and non-DSPN groups and on the EMG parameters of the DSPN and control groups. TSA was performed on all 12 diagnostic parameters that were selected for meta-analysis. Although the pooled effective size did not exceed the RIS, the TSA established sufficient and conclusive evidence. Figures S1 and S2 illustrate the TSA results for NCS and the CCM parameters of the DSPN and non-DSPN groups. The TSA on all NCS and CCM parameters indicated that the cumulative Z-curve crossed the conventional boundary for benefit and the trial sequential monitoring boundary for benefit, demonstrating that the results were robust and conclusive and further studies were not required. TSA exhibited conclusive results for the EMG parameter of the time to obtain the activation peak in the VL muscle (Fig. S3). However, studies on the TA, LG, and GM muscles of the DSPN and control groups were not conclusive because the Z-curves were located between the TSA monitoring boundaries, indicating the involvement of insufficient information in the meta-analysis (Fig. S3). Additional relevant studies are necessary to prove the significance of EMG in the diagnosis of DSPN. Figure S4 shows the TSA results for VPT for the DSPN and non-DSPN groups. The Z-value crossed the TSA monitoring boundaries, indicating that the studies included for meta-analysis were conclusive.

## Discussion

Diabetic Neuropathy (DN), one of the major complications of patients with DM^53^, has attracted the attention of researchers for past few decades. DSPN is the most common distal and symmetrical form of DN. Over the years, a vast range of diagnostic tools for DSPN (symptom scores, QST, and electrophysiology) have been introduced by researchers. The evaluation of DSPN by using clinical assessment instruments is simple and inexpensive, but the obtained results vary during reproduction. Thus, their accuracy remains questionable. According to the position statement of the American Diabetic Association (ADA)^54^, combining clinical history and examination is highly suggested for the clinical diagnosis of DSPN. However, the evaluation of DSPN through clinical history and examination varies due to the lack of standardized baselines. The identification of the appropriate patient population is critical for the valid and careful diagnosis of DSPN in clinical research. The ADA recommended the use of validated clinical instruments combined with electrophysiology and measurements of small-fiber damage and repair obtained via NCS or CCM^55^. Therefore, researchers and health professionals have conducted different clinical studies on DSPN by using different screening methods and, in many cases, two or more methods, to diagnose DSPN accurately^9,10^. Therefore, this work aimed to analyze the existing literature on clinical studies on DSPN to help understand the characteristics of patients and the nature of different screening parameters for control, non-DSPN, and DSPN groups. This review also focused on finding statistically significant relationships among different diagnostic parameters that have been reported in the literature for identifying DSPN.

Three noninvasive electrophysiological methods for the diagnosis of DSPN, i.e., NCS, CCM, and EMG, were considered for this review and meta-analysis because this work aimed to understand the effect of diagnostic parameters in the control, non-DSPN, and DSPN groups. The following diagnostic parameters were considered for meta-analysis and systematic review: PMNCV, PMNamp, SSNCV, and SSNamp for NCS; NFD, NBD, and NFL for CCM; and time to peak occurrence (from 0 to 100% of the stance phase) for VL, TA, LG, and GM muscles for EMG and VPT.

The 19th annual Diabetic Neuropathy Study Group of the European Association for the Study of Diabetes (NEURODIAB) identified NCS as the first objective quantitative indication of DSPN^56^. NCS, a noninvasive method, has been recommended for epidemiologic surveys or controlled clinical trials on DSPN as an early and reliable indicator of the occurrence of this neuropathy^56^. NCS has been used as a standardized method for identifying patients with DSPN and validating the performances of other methods^19,24,26–28,30,46–50^. This meta-analysis revealed that the DSPN group showed reduced NCV and NA in SS and PM nerves compared with the other two experimental groups. This result satisfied and validated the accuracy of the included studies for NCS.

CCM is a new noninvasive method that has been widely used in clinical studies on identifying small-fiber neuropathy^19–31^. CCM involves the use of in vivo images to study the corneal structure in corneal disease identification. Small-fiber DSPN affects sensitive nerve fibers in the human cornea, and CCM has shown good sensitivity in identifying small-fiber DSPN at a very early stage. Many review studies have been conducted to describe different approaches for CCM imaging and observed the clinical correlation of CCM in the assessment of DSPN^15,57,58^. The use of CCM is increasing rapidly given its advantages in the assessment of DSPN at an early stage. Therefore, we considered CCM in our meta-analysis as one of the methods for DSPN diagnosis. The meta-analysis revealed that the CCM parameters of the DSPN group were drastically reduced compared with those of the non-DSPN and control groups.

EMG^55,56,59–62^ is an electrophysiological method that measures the electrical activity of muscles. It^36–40^ has been used to evaluate the change in a muscle’s electrical activity to diagnose DSPN in clinical research. Compared with other groups, the DSPN group showed greater stance phase time^63–65^ and decreased and delayed lower limb muscle activity; in particular, the VL, TA, and GM are the most affected by the progression of neuropathy^38,66^. Thus, EMG^36–40^ is widely used in different clinical research and trials to diagnose DSPN and observe the biomechanics changes in different muscle activities due to DSPN. Akashi et al.^38^ reported that patients with DSPN and ulceration show delayed activation peak in the VL and LG muscles. Gomes et al.^39^ reported delayed activation peak in the VL, TA, GM, and fibularis longus muscles during gait.

In this meta-analysis, the time to peak occurrence (from 0 to 100% of the stance phase) for four lower limb muscles (VL, TA, LG, and GM) were considered as diagnostic parameters for all three experimental groups. All these muscles showed changes in activities due to DSPN. The time of peak muscle activity occurrence was longer for the VL, LG, and GM but reduced for the TA in the DSPN group compared with those in the control group. Our meta-analysis suggested that compared with that in the DSPN and control groups, the peak occurrence in the TA and GM was non-significantly longer and that for VL in the DSPN group did not differ. A meta-analysis was not possible due to lack of studies on the LG muscle of the non-DSPN groups.

All the parameters’ mean values, which were calculated from all the included studies, were subjected to Student’s *t-*test to observe statistically significant differences between the parameters of three diagnostic methods (NCS, CCM, and EMG) for different experimental groups (control, non-DSPN, and DSPN groups). For the DSPN experimental group, PMNCV and NFD (p = 0.29); time to peak occurrence for VL and NFL (p = 0.68); VPT and NFD (p = 0.44); VPT and PMNCV (p = 0.16); and SSNA and time to peak occurrence for TA (p = 0.15) were statistically non-significant. For each experimental group, most of the parameters showed statistically significant difference between each other. However, no specific pattern for statistically non-significant parameter pairs was found among the three different experimental groups. This analysis indicated that the accuracy of the diagnostic methods is doubtable if any two methods with statistically non-significant parameters are considered for the diagnosis of DSPN. Further analysis is required to understand the difference in statistical patterns for all the screening variables among the three experimental groups.

Given that the values of diagnostic parameters changed depending on the different conditions of the patients and environments, obtaining a baseline value for each experimental group can be challenging. All the diagnostic parameter values from the included studies were used to find the summarized value of each parameter. After finding the summarized value of each parameter, meta-analysis was used to identify the heterogeneity of this observation. We conducted our meta-analysis for each parameter of three different diagnostic methods (NCS, CCM, and EMG) that have been widely used for clinical researches on DSPN. The studies included in our meta-analysis on NCS and CCM parameters (all p < 0.001) for DSPN and non-DSPN groups showed good heterogeneity, indicating that the effect of the included studies were acceptable for obtaining a conclusion on the baseline values for each diagnostic parameters. However, for EMG, few studies have reported the time to delay in activation peak for the non-DSPN group. This situation prevented us from conducting a meta-analysis. Therefore, we conducted the meta-analysis on the DSPN and control groups for EMG, and all three lower limb muscles (TA, LG and GM), except for VL, showed good heterogeneity. Moreover, we recommend adding clinical trials for studying patients EMG from lower limbs to understand the change in muscle activity due to DSPN. The time to muscle activation peak in DSPN and control group for the TA muscle was not statistically significant, and the meta-analysis exhibited low heterogeneity for the VL and GM muscles. Meta-analysis on the included studies reporting VPT revealed moderate heterogeneity, indicating that studies must be added to obtain robust conclusion. Good heterogeneity indicates that the included studies have variations in the data and that the baseline values calculated from the included studies can be considered as reliable standardized values.

A number of reviews have been conducted on different diagnostic methods of DSPN. Jiang et al.^8^ conducted a meta-analysis on CCM for the assessment of DSPN. They found that all the CCM parameters, except for nerve fiber tortuosity coefficient, were significantly reduced in the DSPN group relative to that in the control and non-DSPN groups. In our meta-analysis, we also observed that all the CCM parameters decreased in the DSPN group. Fernando et al.^45^ reviewed the biomechanical characteristics of DSPN. Although they considered the EMG dynamics of the three studies, in their meta-analysis, they only observed the TA muscle of the DSPN and control group. We considered five studies and the time to activation peak occurrence of four lower limb muscles in three different experimental groups. Li et al.^67^ observed the correlation among three diabetic microvascular diseases, namely, DN, diabetic retinopathy, and diabetic kidney disease, but did not focus on diagnostic methods. One drawback of all these studies is that they did not conduct TSA to verify the conclusiveness of their meta-analysis. Shabeeb et al.^68^ systematically reviewed electrophysiological examinations for DSPN. They summarized the list of studies using NCS and EMG diagnostic methods reported over 2008 to 2018 and suggested the use of electrophysiological studies for the assessment of DSPN. However, their study have not conducted any meta-analysis. To the best of our knowledge, this is the first study that have conducted meta-analysis with trial sequential analysis and observed statistically significant differences among noninvasive electrophysiological methods for the assessment of DSPN.

TSA was conducted to validate the meta-analysis and hence prove the validity of the calculated baseline values of each diagnostic parameter for three experimental groups. TSA is used to decide if the results from any meta-analysis are conclusive or not. All the 12 diagnostic parameters that were selected for meta-analysis were subjected to TSA. For NCS, CCM and VPT parameters, TSA has been observed for DSPN and non-DSPN groups. Studies involving DSPN and control groups were considered for the TSA of EMG parameters. Although the pooled effective size did not exceed the RIS, TSA established sufficient and conclusive evidence and indicated that no further observational trials are required for NCS and CCM parameters, the meta-analysis depicted conclusive observational evidence, and the analytical findings are sufficiently robust as baseline values for future studies. However, the results for the EMG parameters of four different muscles were inconclusive, and additional trials are needed to understand the effect of DSPN on lower limb muscles. TSA results for VPT were conclusive. Moderate heterogeneity from the meta-analysis and a conclusive TSA for VPT indicated that even though the included studies exhibited visible difference in the data of patient groups, additional studies should be included in the meta-analysis to obtain a solid conclusive result.

One major limitation of this study is that only five study have been found in the literature those have considered lower limb EMG to investigate change in muscle activity due to DSPN as a diagnosis criteria, which leads to the poor heterogeneity, and inconclusive meta-analysis for EMG parameters. Additional studies must be conducted to observe the effect of time to peak occurrence in lower limb muscles due to the progression of DSPN. Another drawback of this study is all the sensory and motor nerve parameters should be studied to understand the propagation of DSPN in different nervous systems. In conclusion, this systematic review and meta-analysis considered a larger sample size for each diagnostic method than individual studies. The results for NCS and CCM showed that the included studies had potentially variable data, and the meta-analysis showed good heterogeneity. This study can be a have a promising effect for the upcoming research work to understand the effect of the three noninvasive electrophysiological methods on DSPN identification.

## Supplementary Information

Supplementary Information.

## Abbreviations

ADA: American Diabetic Association
BMI: Body Mass Index
CCM: Corneal confocal microscopy
CI: Confidence interval
DN: Diabetic neuropathy
DSPN: Diabetic sensorimotor polyneuropathy
EMG: Electromyography
FL: Fibularis longus
GM: Gastrocnemius medialis
HbA1c: Glycated hemoglobin
IENF: Intra-epidermal nerve fiber
LG: Lateral gastrocnemius
NCV: Nerve conduction velocity
NCS: Nerve conduction studies
NA: Nerve amplitude
NFD: Nerve fiber density
NBD: Nerve branch density
NFL: Nerve fiber length
PM: Peroneal motor
PMNCV: Peroneal motor nerve conduction velocity
PMNamp: Peroneal motor nerve amplitude
QST: Quantitative sensory testing
RIS: Required information size
SD: Standard deviation
SMD: Standard mean difference
SS: Sural sensory
SSNCV: Sural sensory nerve conduction velocity
SSNamp: Sural sensory nerve amplitude
TA: Tibialis anterior
TSA: Trial sequential analysis
VPT: Vibration perception threshold
VL: Vastus lateralis
