# Supplementary material for: Performance analysis of noninvasive electrophysiological methods for the assessment of diabetic sensorimotor polyneuropathy in clinical research: a systematic review and meta-analysis with trial sequential analysis
Source: Sci Rep. 2020 Dec 10;10:21770. doi: 10.1038/s41598-020-78787-0 (PMC7730399; doi:10.1038/s41598-020-78787-0)
Supplement: Supplementary file 1 — Supplementary Information. [file 41598_2020_78787_MOESM1_ESM.docx]

**Performance analysis of noninvasive electrophysiological methods for the assessment of diabetic sensorimotor polyneuropathy in clinical research: A systematic review and meta-analysis with trial sequential analysis**

Fahmida Haque^1^, Mamun Bin Ibne Reaz^1, *^, Sawal Hamid Md Ali^1^, Norhana Arsad^1^, Muhammad Enamul Hoque Chowdhury^2, *^

^1^Department of Electrical, Electronic and System Engineering, Universiti Kebangsaan Malaysia, Bangi, 43600, Selangor, Malaysia.

^2^Department of Electrical Engineering, Qatar University, Doha, 2713, Qatar

^*^Corresponding author: [mamun@ukm.edu.my](mailto:mamun@ukm.edu.my); [mchowdhury@qu.edu.qa](mailto:mchowdhury@qu.edu.qa)


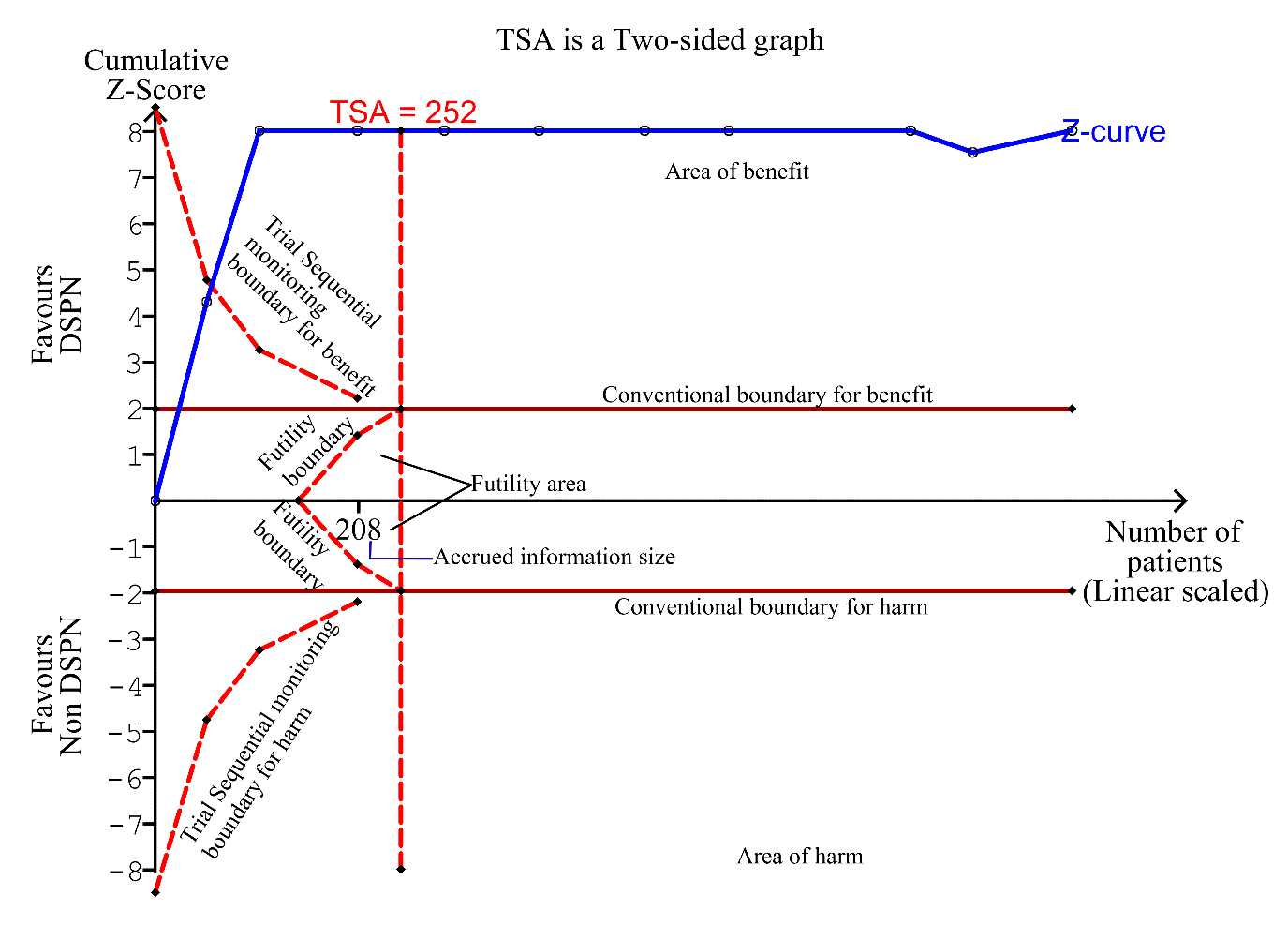


(a)


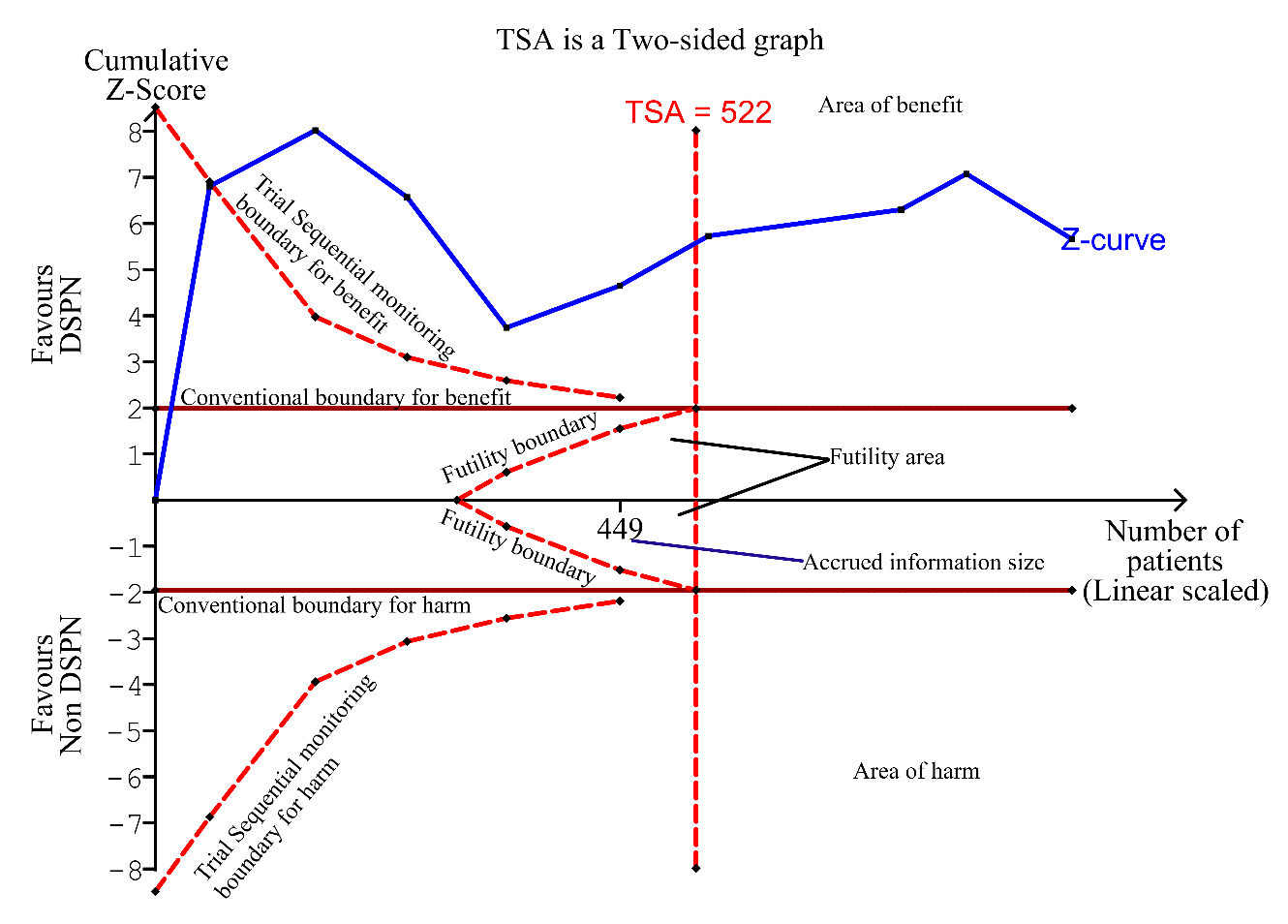
 (b)


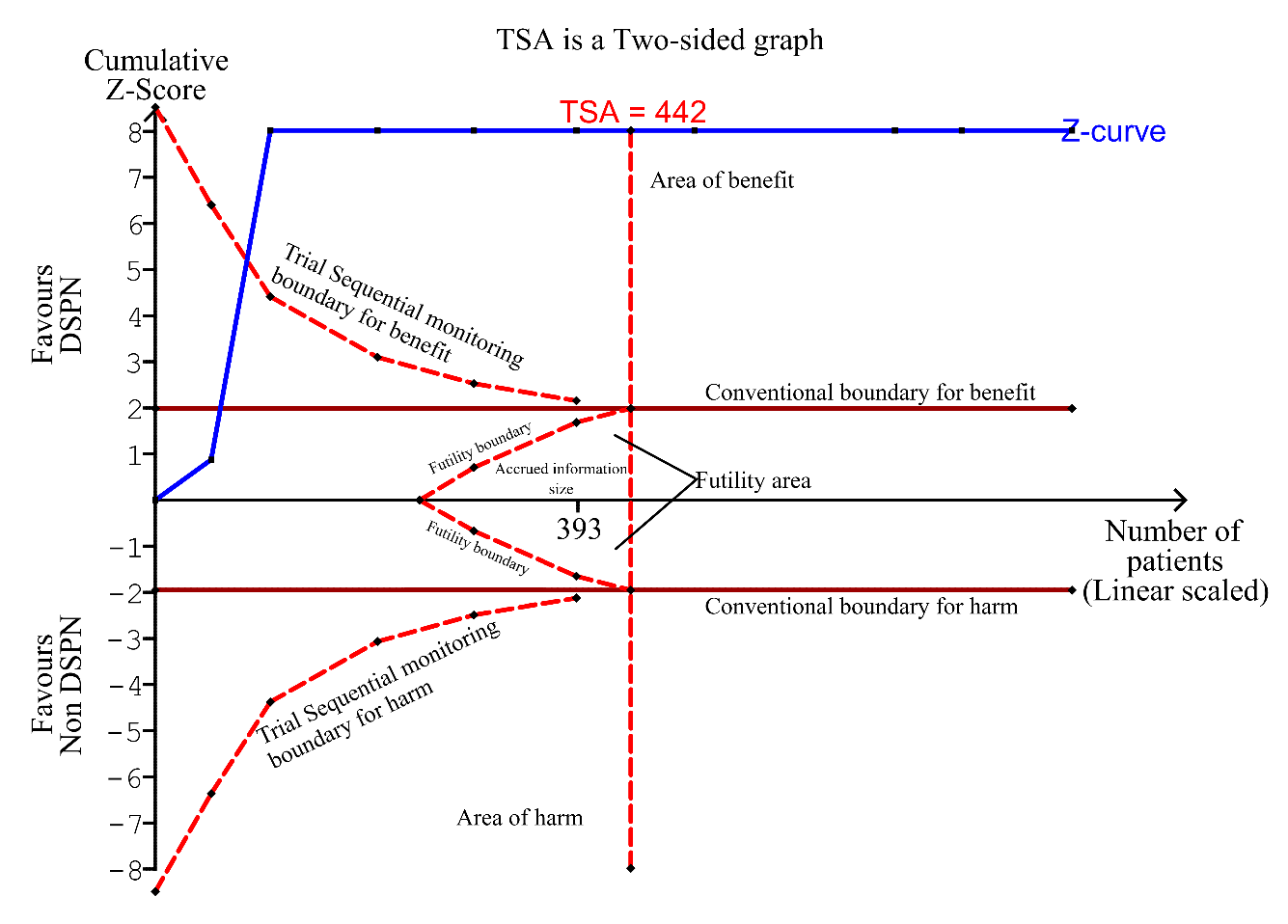


(c)


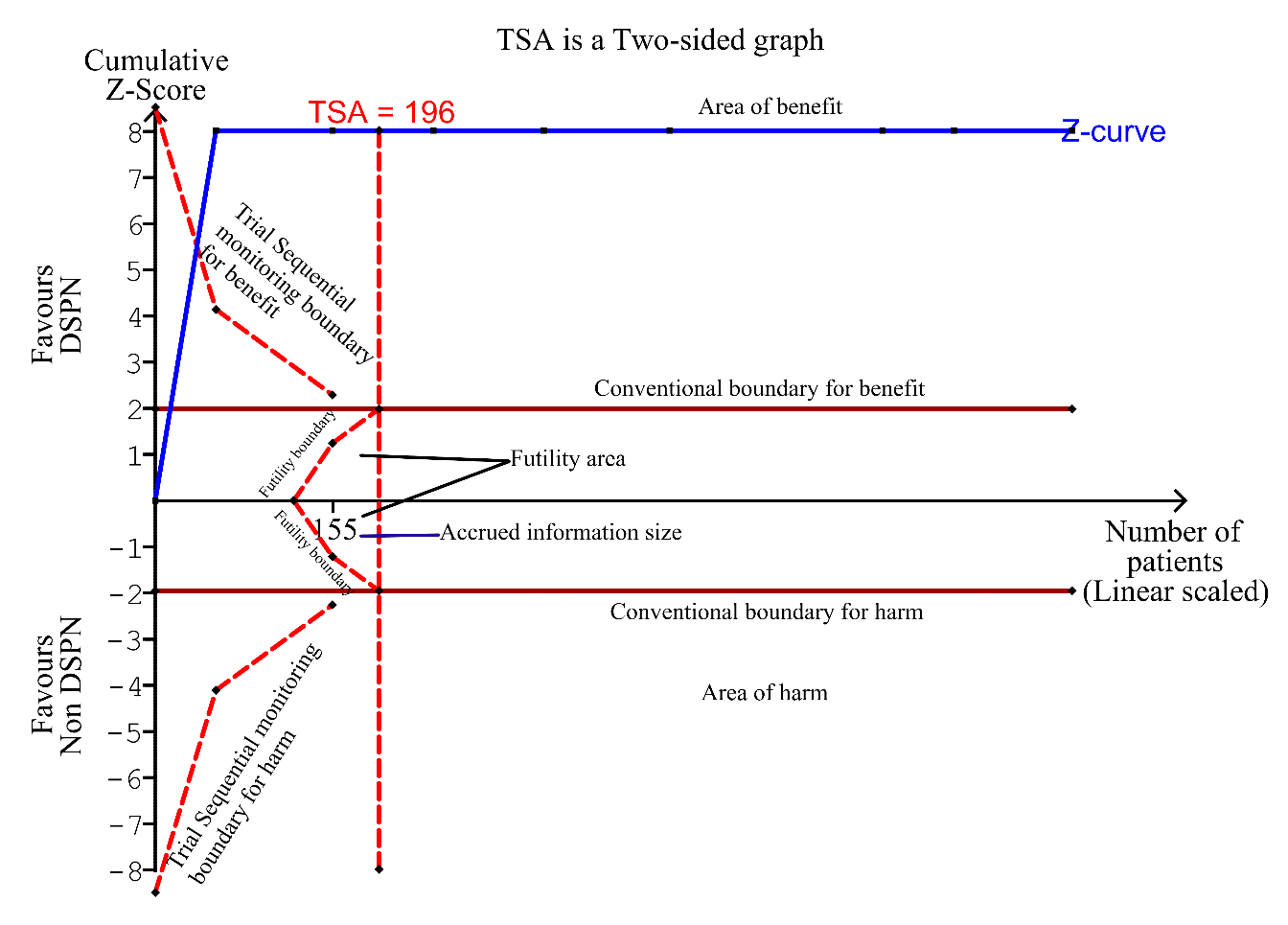


(d)

Figure S1: Trial Sequential Analysis (TSA) of NCS diagnosis parameters comparing DSPN and Non-DSPN groups (a) PMNCV (b) SSNCV (c) PMNamp (d) SSNamp.


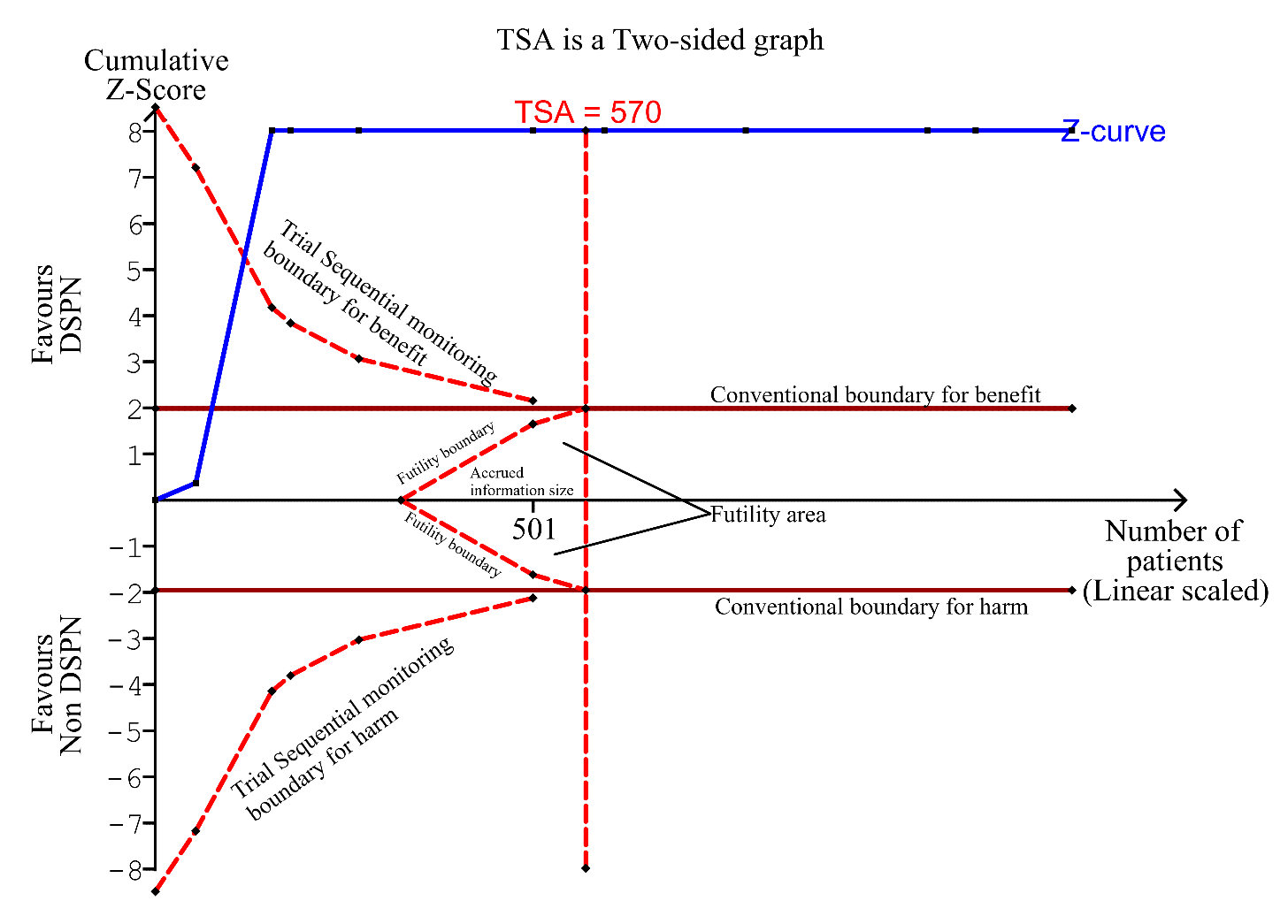


(a)


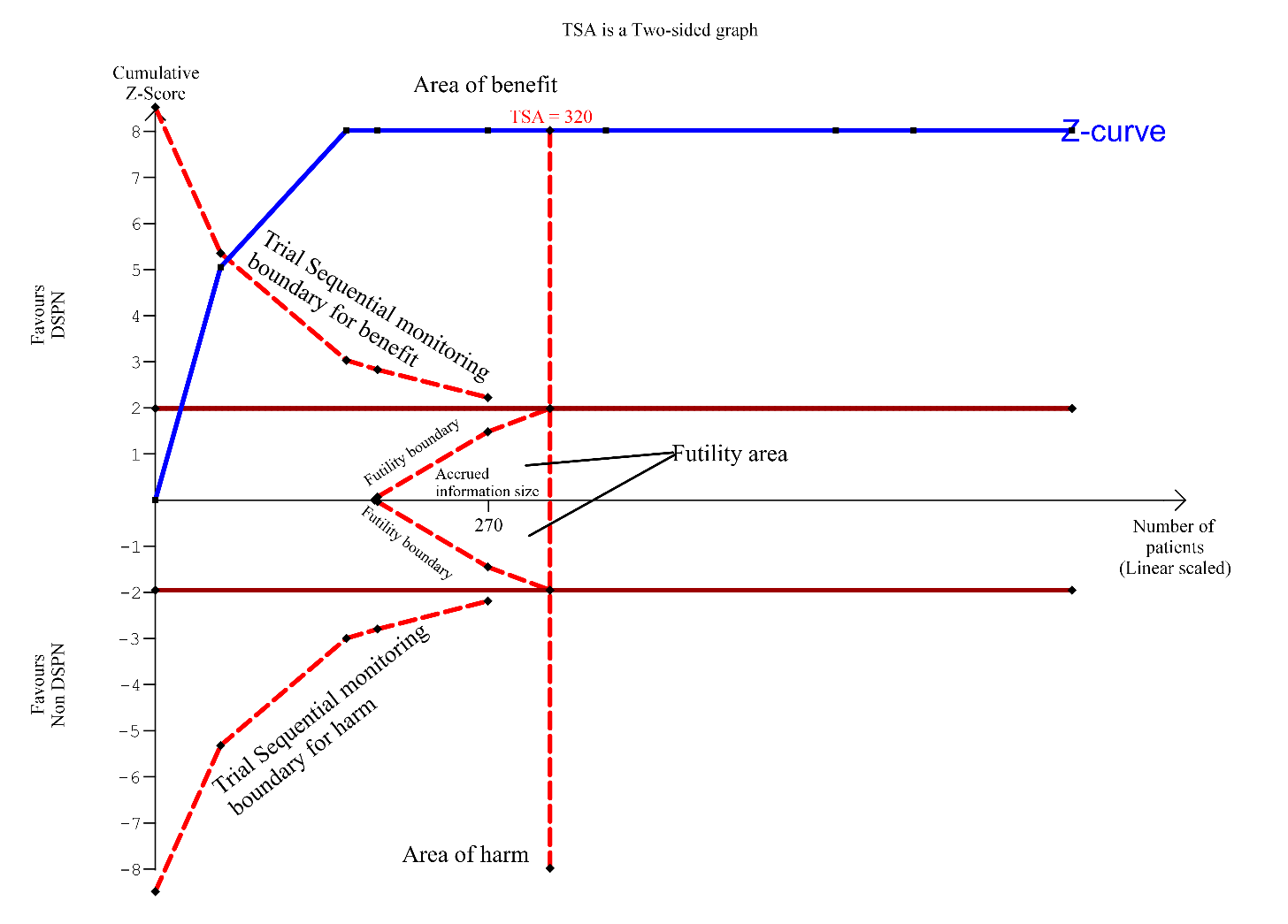


(b)


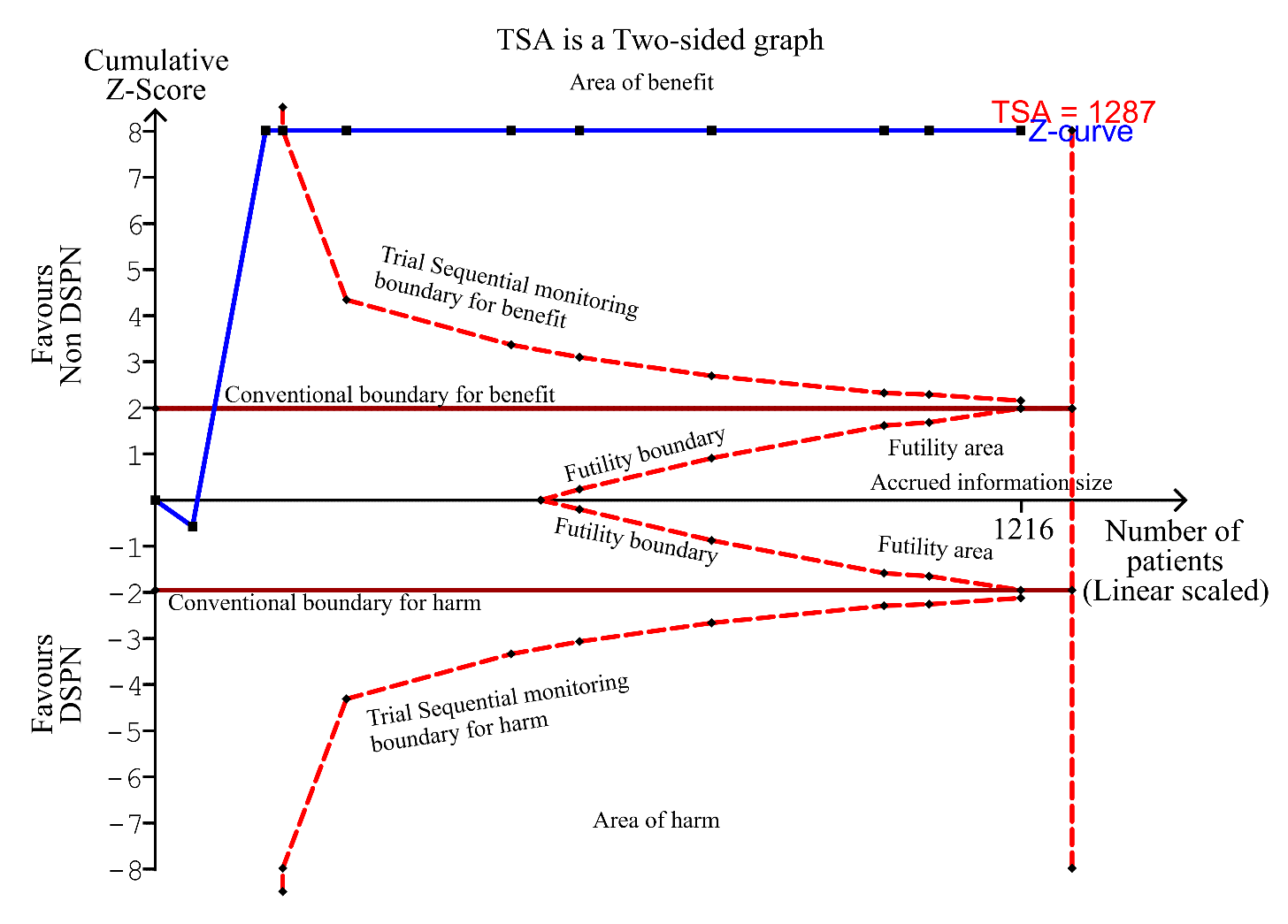


(c)

Figure S2: Trial Sequential Analysis (TSA) of CCM diagnosis parameters comparing DSPN and Non-DSPN groups (a) CNFL (b) CNFD (c) CNBD.


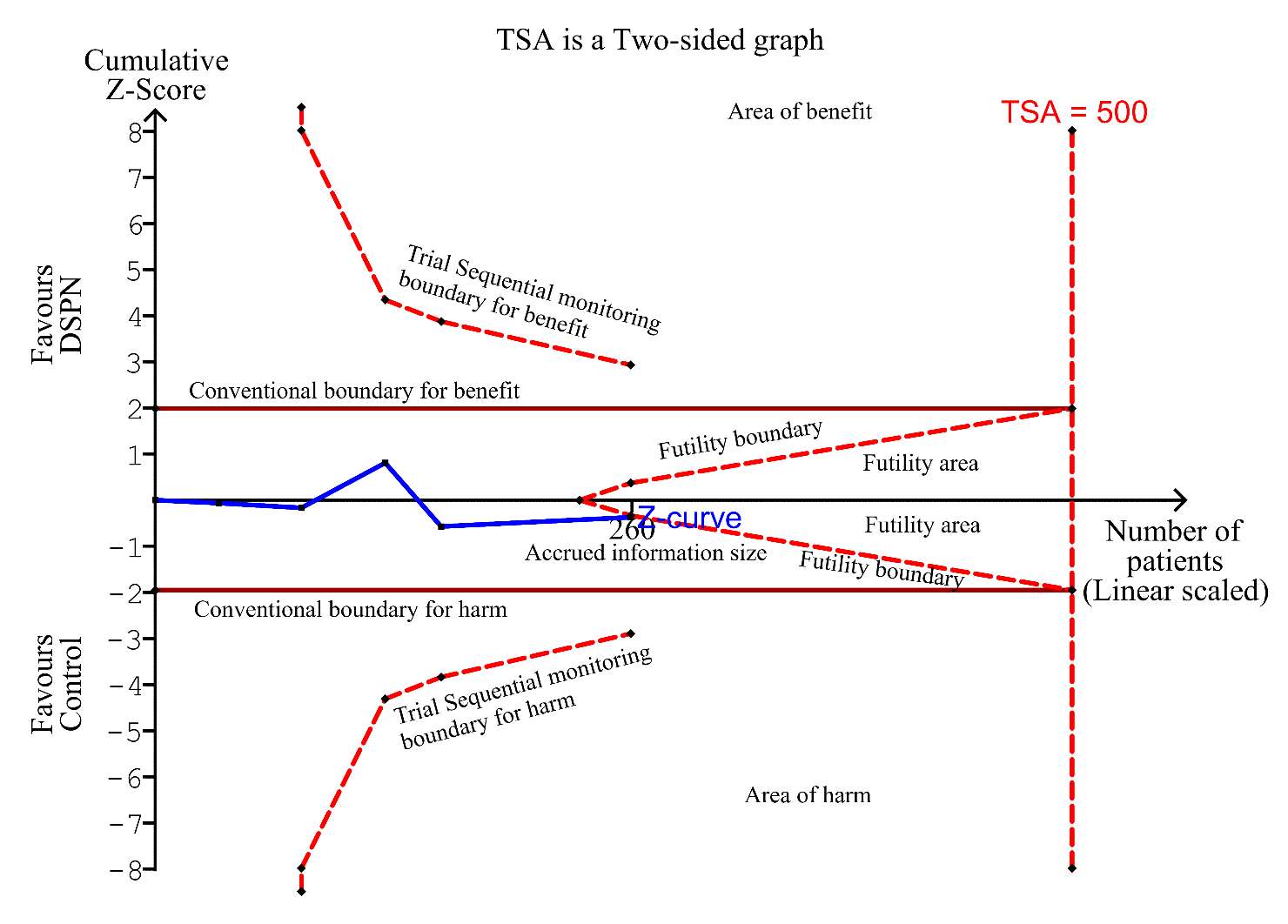


(a)


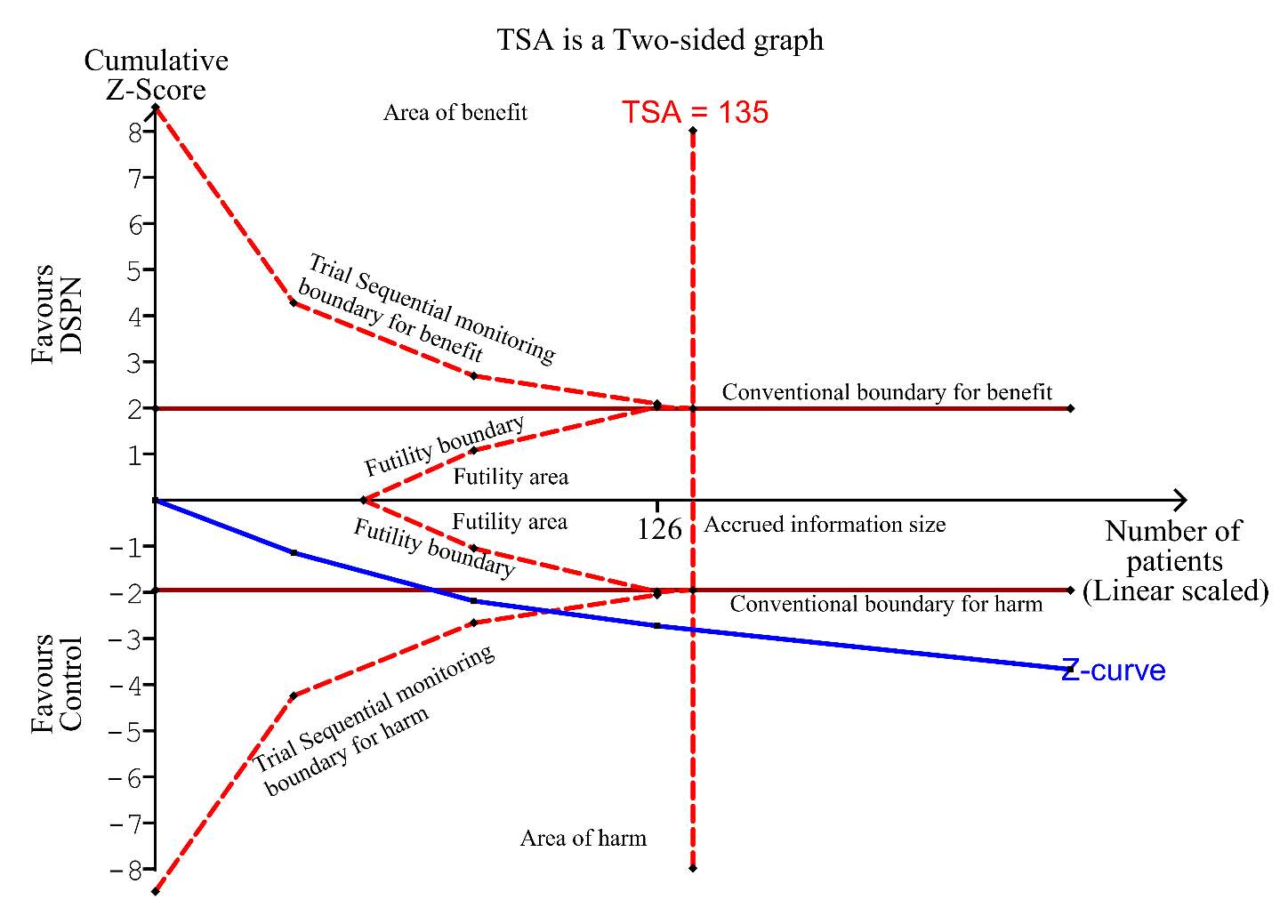


(b)


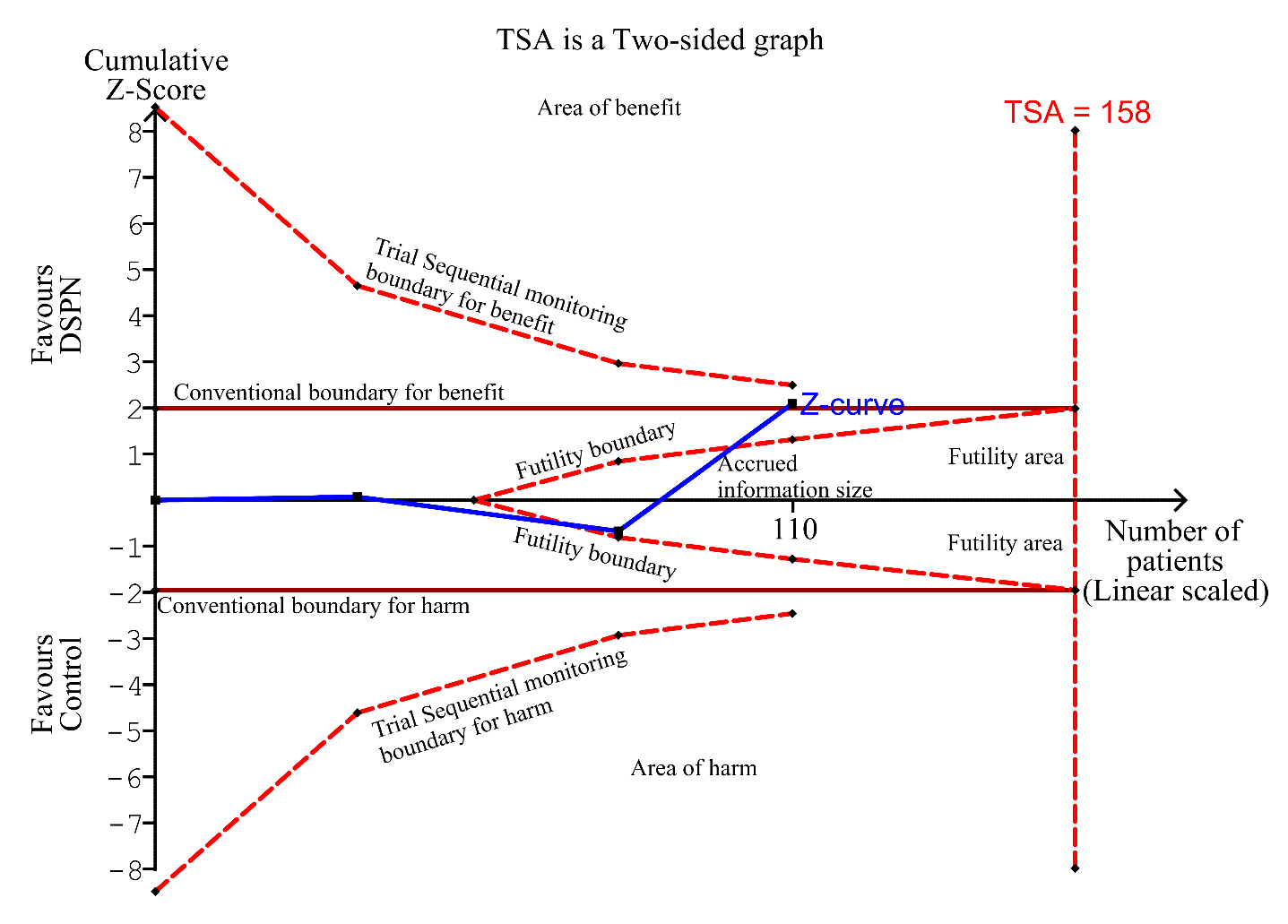


(c)


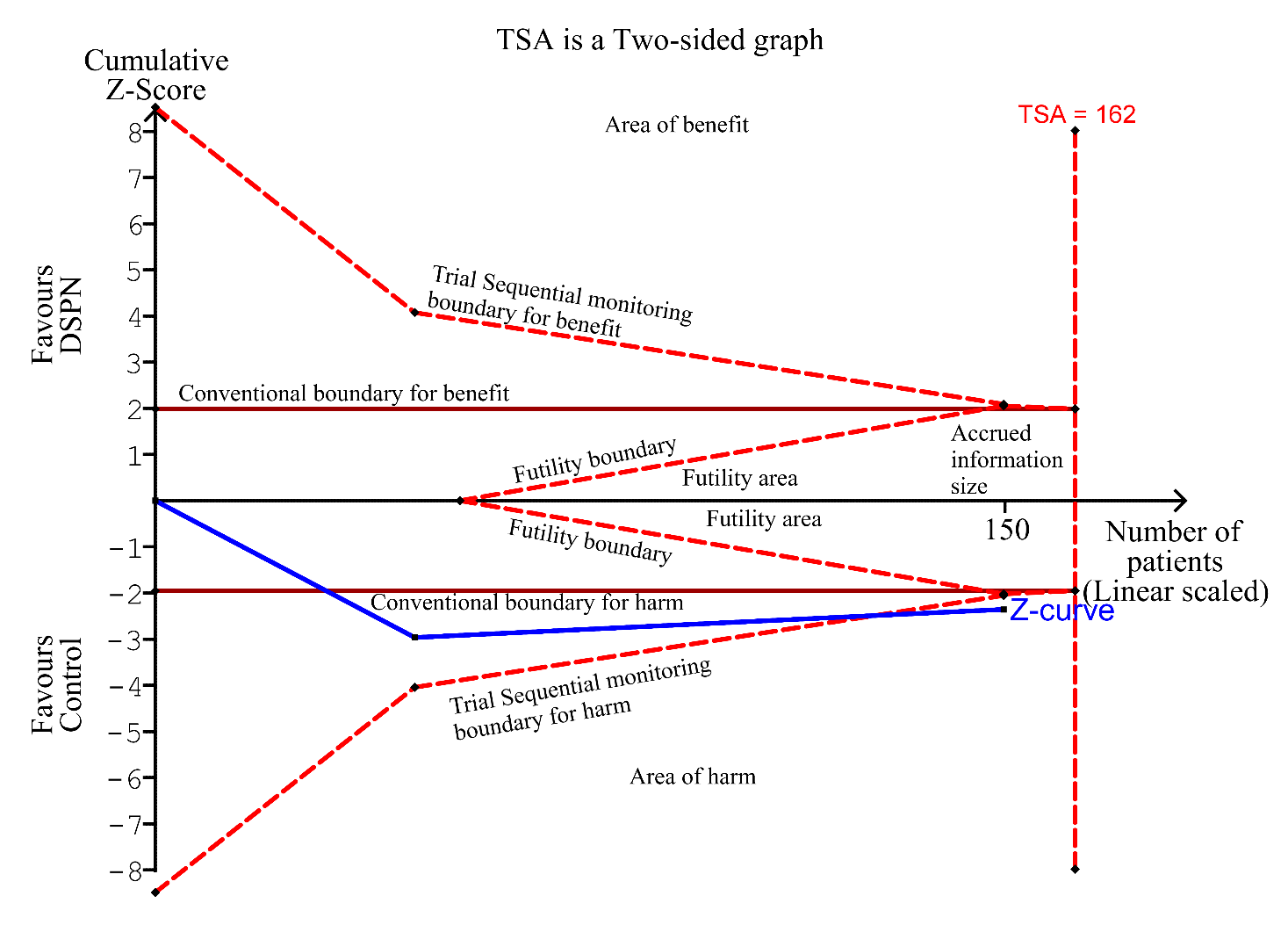


(d)

Figure S3: Trial Sequential Analysis (TSA) of EMG parameters of time for muscle activation peak for four different lower limb muscles(a) TA (b) VL (c) LG (d) GM for DSPN and control groups


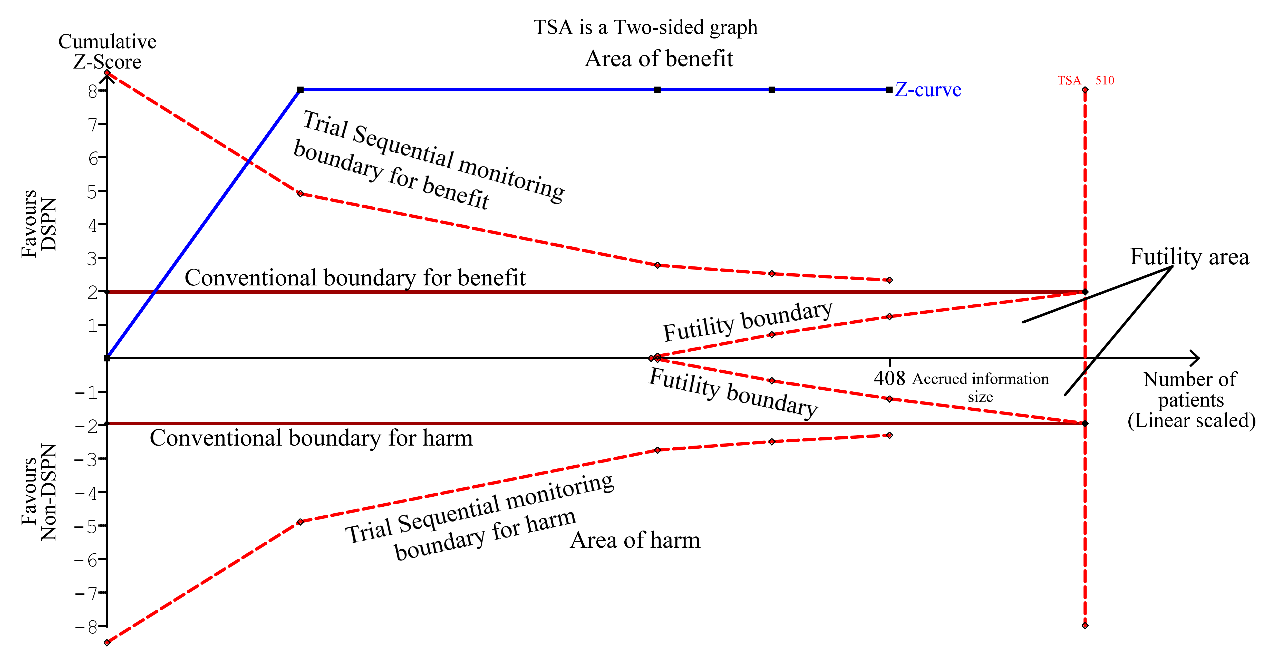


Figure S4: TSA of VTP for DSPN and Non-DSPN groups.

Table S1: Statistical Significance between different diagnostic parameters for Control group.

|  | | NFL | NFD | NBD | PMNCV | SSNCV | SSNamp | PMNamp | VPT | Time of peak occurrence (%) | | | |
| --- | --- | --- | --- | --- | --- | --- | --- | --- | --- | --- | --- | --- | --- |
|  |  |  |  |  |  |  |  |  |  | VL | TA | GM | LG |
| NFL | | / |  |  |  |  | 0.45 |  |  |  |  |  |  |
| NFD | |  | / | 0.0005 | 0.0002 | <0.0001 |  |  |  |  |  |  | 0.37 |
| NBD | |  | 0.0005 | / |  |  |  |  |  |  |  |  | 0.0008 |
| PMNCV | |  | 0.0002 |  | / | 0.082 |  |  |  |  |  |  |  |
| SSNCV | |  |  |  | 0.082 | / |  |  |  |  |  |  |  |
| SSNamp | | 0.45 |  |  |  |  | / |  |  |  |  |  |  |
| PMNamp | |  |  |  |  |  |  | / | 1.00 |  | 0.0003 |  |  |
| VPT | |  |  |  |  |  |  | 1.00 | / | 0.004 | 0.048 |  |  |
| Time of peak occurrence (%) | VL |  |  |  |  |  |  |  | 0.0047 | / |  |  |  |
|  | TA |  |  |  |  |  |  | 0.0003 | 0.048 |  | / |  |  |
|  | LG |  |  |  |  |  |  |  |  |  |  | / |  |
|  | GM |  | 0.37 | 0.0008 |  |  |  |  |  |  |  |  | / |

The blank spaces indicate significance value p<0.0001

Table S2: Statistical Significance between different diagnostic parameters for Non-DSPN group.

|  | | NFL | NFD | NBD | PMNCV | SSNCV | SSNamp | PMNamp | VPT | Time of peak occurrence (%) | | | |
| --- | --- | --- | --- | --- | --- | --- | --- | --- | --- | --- | --- | --- | --- |
|  |  |  |  |  |  |  |  |  |  | VL | TA | GM | LG |
| NFL | | / |  |  |  |  | 0.00051 |  |  |  |  |  |  |
| NFD | |  | / | 0.00013 | 0.0008 |  |  |  |  |  |  |  |  |
| NBD | |  | 0.00013 | / |  |  |  |  |  |  |  |  |  |
| PMNCV | |  | 0.00013 | 1.00 | / |  |  |  |  |  |  |  |  |
| SSNCS | |  |  |  | 0.1203 | / |  |  |  |  |  |  |  |
| SSNamp | | 0.00051 |  |  |  |  | / |  | 0.0003 | 0.71 |  |  | 0.47 |
| PMNamp | |  |  |  |  |  |  | / | 0.28 |  | 0.16 |  |  |
| VPT | |  |  |  |  |  | 0.0003 | 0.28 | / |  |  |  |  |
| Time of peak occurrence (%) | VL |  |  |  |  |  | 0.71 |  |  | / |  |  | 0.47 |
|  | TA |  |  |  |  |  |  | 0.16 | 0.0003 |  | / |  |  |
|  | GM |  |  |  |  |  |  |  |  |  |  | / |  |
|  | LG |  |  |  |  |  | 0.47 |  |  | 0.47 |  |  | / |

The blank spaces indicate significance value p<0.0001

Table S3: Statistical Significance between different diagnostic parameters for DSPN group.

|  | | NFL | NFD | NBD | PMNCV | SSNCV | SSNamp | PMNamp | VPT | Time of peak occurrence (%) | | | |
| --- | --- | --- | --- | --- | --- | --- | --- | --- | --- | --- | --- | --- | --- |
|  |  |  |  |  |  |  |  |  |  | VL | TA | LG | GM |
| NFL | | / |  | 0.0087 |  |  |  |  |  | 0.68 |  |  | 0.0002 |
| NFD | |  | / |  | 0.29 |  |  |  | 0.44 |  |  |  |  |
| NBD | | 0.00876 |  | / |  |  |  |  | 0.04 | 0.01 |  |  |  |
| PMNCV | |  | 0.29 |  | / | 0.022 |  |  | 0.16 |  |  |  |  |
| SSNCS | |  |  |  | 0.022 | / |  |  | 0.002 |  |  |  |  |
| SSNamp | |  |  |  |  |  | / | 0.007 |  |  | 0.15 |  | 0.004 |
| PMNamp | |  |  |  |  |  | 0.007 | / |  |  | 0.004 |  |  |
| VPT | |  | 0.44 | 0.04 | 0.16 | 0.002 |  |  | / |  |  |  |  |
| Time of peak occurrence (%) | VL | 0.68 |  | 0.014 |  |  |  |  |  | / |  |  |  |
|  | TA |  |  |  |  |  | 0.15 | 0.004 |  |  | / |  |  |
|  | LG |  |  |  |  |  |  |  |  |  |  | / |  |
|  | GM | 0.0002 |  |  |  |  | 0.004 |  |  |  |  |  | / |

The blank spaces indicate significance value p<0
